# Multifunctional antibody responses from a primate *Shigella* outbreak inform vaccine design

**DOI:** 10.1126/scitranslmed.aef7084

**Published:** 2026-09-02

**Authors:** Robert M. Gallant, Paul Savarino, Sophia Pulido, Morgan S.A. Gilman, Ti Lu, Jennifer Hayes, Nicholas L. Xerri, Torrey Williams, Mark A. Ochoa, Zackary Dietz, Eric Peterson, Timothy Scott, Faye Hartmann, Stephen G. Baker, Robert W. Kaminski, Devin Sok, Andrew C. Kruse, Wendy Picking, Saverio V. Capuano, Hayden R. Schmidt

**Affiliations:** 1 IAVI Neutralizing Antibody Center, San Diego, California, USA; 2 Department of Biological Chemistry and Molecular Pharmacology, Blavatnik Institute, Harvard Medical School, Boston, Massachusetts, USA; 4 Bond Life Sciences Center and Department of Veterinary Pathobiology, University of Missouri, Columbia, Missouri, USA; 5 Wisconsin National Primate Research Center, University of Wisconsin-Madison, Madison, Wisconsin, USA; 6 Department of Medicine, Cambridge Institute for Therapeutic Immunology and Infectious Disease, Jeffrey Cheah Biomedical Centre, University of Cambridge, Cambridge, UK; 7 UW Veterinary Care, University of Wisconsin-Madison, Madison, Wisconsin, USA; 8 IAVI UK, London, UK; 9 Latham Biopharm Group, Cambridge, Massachusetts, USA

## Abstract

There is currently no approved vaccine for *Shigella* spp., a leading cause of diarrhea with increasing rates of antimicrobial resistance. *Shigella* vaccine development is complicated in part by an incomplete understanding of the structural and molecular determinants of immunity. To address this, we isolated monoclonal antibodies against candidate *Shigella* vaccine antigens using samples from a *Shigella flexneri* outbreak in a nonhuman primate (NHP) research facility. We found that antibodies targeting the *Shigella* O-antigen can undergo substantial affinity maturation (>10%) to acquire broad cross-reactivity across *S. flexneri* serotypes. We also found that the virulence-associated type III secretion system (T3SS) proteins IpaD and IpaB elicit moderate T cell and robust antibody responses. T3SS antibodies could either inhibit or enhance bacterial virulence in vitro and differed in vivo depending on their epitope specificity. Collectively, these findings provide insights into protective and deleterious immune responses against *Shigella* that directly inform vaccine immunogen design.

## Introduction

*Shigella* infection is a leading cause of diarrheal disease, accounting for over 200,000,000 cases globally each year (1). These cases result in over 200,000 deaths and substantial morbidity among young children (2). As rates of resistance to antimicrobials increase among *Shigella* isolates (3, 4), an effective vaccine is increasingly important. Several *Shigella* vaccine candidates are in the pipeline at various stages of development (5). Given that immunity against *Shigella* spp. tends to only protect against a narrow subset of the many clinically relevant serotypes (6), the bacteria are highly virulent (7), and *Shigella* overwhelmingly impacts resource-constrained settings (8), vaccines must meet challenging standards with respect to breadth, potency, and cost of goods to be adopted (9).

One method for maximizing vaccine efficacy is to apply rational antigen design principles (10). These approaches have been effective for the design of vaccines against otherwise intractable pathogens of global health interest, including respiratory syncytial virus (RSV) (11) and severe acute respiratory syndrome coronavirus 2 (SARS-CoV-2) (12). However, rational design of vaccine antigens requires a detailed molecular and structural understanding of both the antigen and the protective host immune response, which is currently lacking for key *Shigella* antigens. Although there is strong evidence that immune responses against O-antigen (O-Ag; the polysaccharide component of lipopolysaccharide that determines *Shigella* serotype (13–17)) and type III secretion system (T3SS) proteins such as IpaB, IpaC, and IpaD (18–20) are relevant for protection against disease, the precise components of the adaptive immune response that mechanistically drive protection remain unidentified.

In December 2022, the Wisconsin National Primate Research Center (WNPRC) began experiencing a high incidence of *Shigella* infection among their nonhuman primates (NHPs), with over 150 animals experiencing symptomatic infection over a two-year period. Although the scale of this outbreak is unusual, its occurrence is not; NHPs are natural hosts for *Shigella*, and outbreaks in zoos (21, 22) and research facilities (23–25) are well-documented. Although *S. sonnei* predominates in human infections in high-income countries, *S. flexneri* is more frequently reported in captive NHP outbreaks, potentially reflecting ecological differences related to sanitation and transmission dynamics (26, 27). This outbreak provided an opportunity to study the immune response to *Shigella* infection under natural exposure conditions in a controlled environment. Here, we combined bacterial genomics, serologic profiling, functional antibody assays, monoclonal antibody (mAb) discovery, T cell functional assays, and structural biology to define pathogen and host immune features associated with *Shigella* infection in this NHP population. By deeply profiling naturally elicited antibodies, this work addresses a central barrier to *Shigella* vaccine development: the need to identify antibody features that confer broad protection while avoiding ineffective or even detrimental immune responses.

## Results

### Subhead 1: The WNPRC outbreak was driven by two genetically distinct clusters of *S. flexneri*

**Figure 1:**
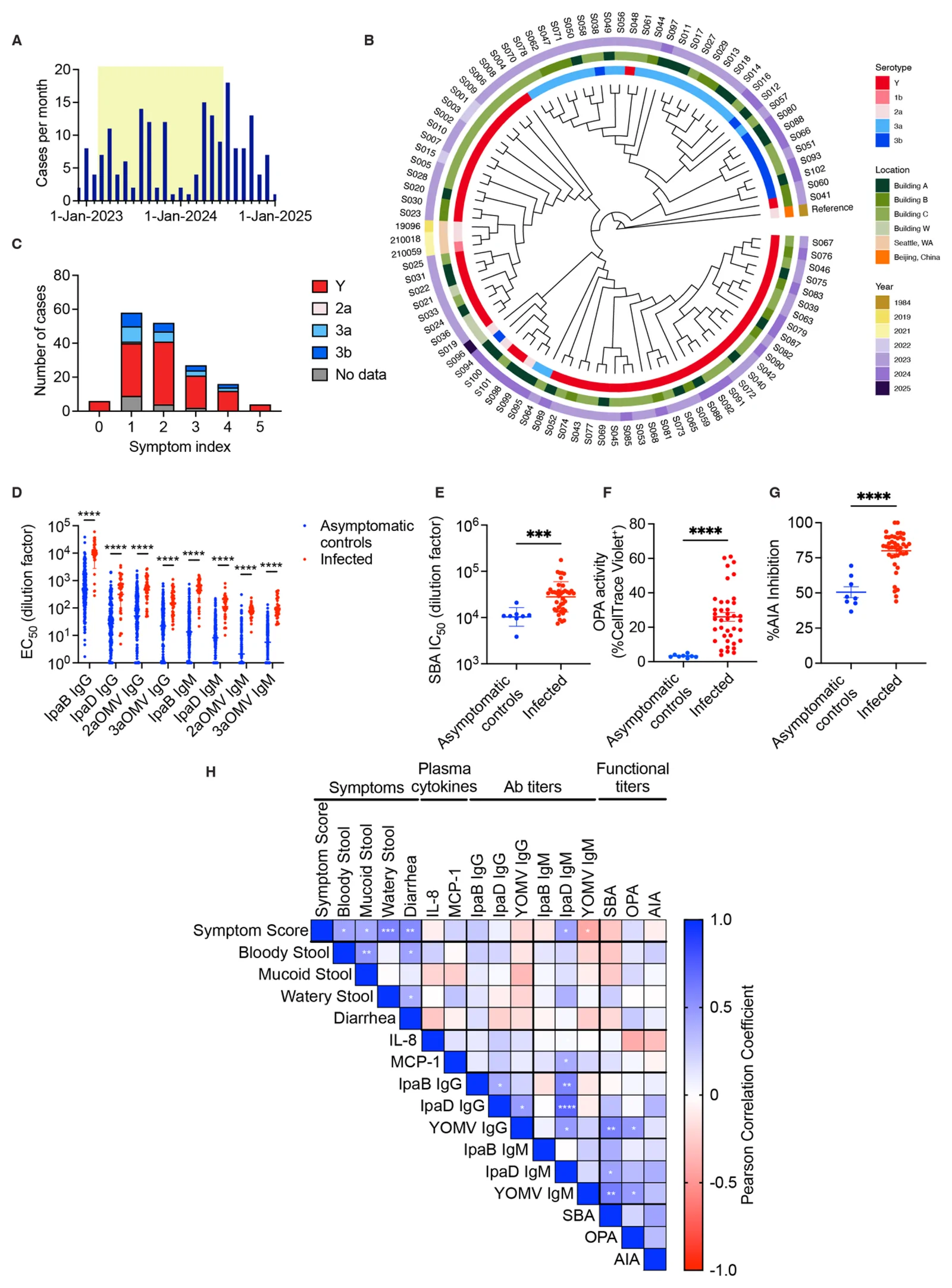
A *Shigella flexneri* outbreak in an NHP research facility induced robust immune responses. (**A**) Timeline of *Shigella* cases in the NHP research facility. Yellow shading indicates the sampling period for this study. (**B**)MaxPhylogenetic cladogram of *S. flexneri* isolates from the outbreak (S###), recent human cases (19096, 210018, and 210059) (34), and the reference strain (301) (35). This tree is shown as a cladogram with branch lengths not proportional to evolutionary distance. Metadata (serotype, location of origin, year of collection) are shown circumferentially. (**C**) Distribution of symptom severity by serotype. 0 indicates asymptomatic infection, 1 to 4 indicates increasing severity, and 5 indicates euthanasia was elected or the animal was found dead. Refer to the Methods section for a detailed explanation of symptom scoring. (**D**) IgG and IgM ELISA EC_50_ values against IpaB, IpaD, 2a OMVs, and 3a OMVs of convalescent serum collected two weeks post-detection of infection (n = 41) and an asymptomatic observational cross-section of the research facility (n = 213). Samples without measurable EC_50_ values were assigned a value of 1. Data are presented as geometric mean ± geometric SD and were analyzed by multiple lognormal Welch’s t-tests with Holm-Šídák correction. Experiments were conducted in technical duplicate. (**E to G**) Functional titers of asymptomatic controls (n = 8) and two weeks post-detection of infection serotype Y-convalescent plasma (n=39). Serum bactericidal assay (SBA) IC_50_ values (E), opsonophagocytosis assay (OPA) activity (% CellTrace Violet^+^) (F), and % adhesion/invasion assay (AIA) inhibition (G). SBA data are presented as geometric mean ± geometric SD, analyzed by lognormal Welch’s t-test. OPA and AIA data are presented as mean ± SEM, analyzed by Welch’s t-tests. All experiments were conducted in technical duplicate. (**H**)Pearson correlation matrix of symptoms, plasma cytokines, serum antibody titers, and serum functional titers of samples collected two-weeks post detection of infection with serotype Y. Plasma cytokines, serum antibody titers, and SBA IC_50_ values were log-transformed. *P < 0.05, **P < 0.01, ***P < 0.001, and ****P < 0.0001.

From December 2022 to January 2025, there were 169 cases of microbiologically confirmed shigellosis in the primate colony (Fig. 1A). We performed classical serotyping on 151 isolates and whole-genome sequencing on 95 representative isolates to assess the phylogenetic diversity of the outbreak. Two main clusters of *Shigella flexneri* were identified: one predominantly serotype Y and the other predominantly serotype 3a/3b (Fig. 1B and fig. S1A). The serotype Y cluster was detected across four colony buildings and the serotype 3a/3b cluster was detected across three buildings, suggesting inter-building spread (Fig. 1B). These isolates were closely related to human *S. flexneri* isolates from a 2019–2021 outbreak in Seattle (28), and more distantly related to the *S. flexneri* 301 reference strain from Beijing, China, 1984 (29) (Fig. 1B and fig. S1A). Some WNPRC staff members developed shigellosis, possibly due to exposure to the infected animals in the NHP colony (personal communication), underscoring the clinical relevance of these isolates. Of the 90 sequenced isolates tested for antimicrobial susceptibility, 57 (63%) were multidrug resistant (MDR), being resistant to ampicillin, amoxicillin, cephalexin, chloramphenicol, and doxycycline (fig. S1B). These MDR isolates were mostly within the serotype Y cluster, and predominantly carried the resistance genes *bla*OXA-1, *cat*A1, *tet*(B), and *aad*A1 (fig. S1B). The serotype Y strains observed in this study were derived from a serotype 2a lineage carrying frameshift mutations in *gtrII*, which converts serotype 2a to serotype Y (30) (fig. S1C).

Symptoms were scored using a symptom index that accounted for the number and severity of diarrhea-associated symptoms in animals that tested positive for *Shigella*. Regardless of infecting serotype, most NHP cases were associated with mild symptoms (a symptom index score of 1 or 2), but a minority of infections were asymptomatic or caused severe disease (Fig. 1C and fig. S2A). Symptom severity did not differ by sex (fig. S2B), though there was a modest positive correlation with age (fig. S2C). Additionally, infants accounted for only 12% of cases but 50% of fatal outcomes, suggesting increased susceptibility to severe disease in young primates, consistent with human epidemiological data (31) (fig. S2C).

### Subhead 2: *S. flexneri* infection elicits innate and humoral immune responses in NHPs

We collected serum, plasma, and peripheral blood mononuclear cells (PBMCs) from 41 infected animals at one, two, and four-to-six weeks post detection of infection. We first measured plasma cytokines one week post detection of infection. Infected primates showed mild increases in IL-8 (*P <* 0.001) and MCP-1 (*P <* 0.05) (fig. S2D), which is consistent with high secretion of IL-8 and MCP-1 following *Shigella* infection in various model organisms (32–34). We observed no changes in other cytokines compared to asymptomatic controls (fig. S2D). We next compared IgG and IgM serum titers two weeks post detection of infection against IpaB, IpaD, and outer membrane vesicles (OMVs) from serotypes 2a and 3a, relative to a cross-sectional cohort of 213 asymptomatic NHPs (of unknown *Shigella* infection status) across the facility. Eight serum samples were considered polyreactive and thus excluded from antibody titer analyses due to high binding to the nonspecific control protein ovalbumin (fig. S2E). Titers against all antigens were significantly (*P <* 0.0001) elevated in infected animals (Fig. 1D). There were several asymptomatic NHPs without a history of diarrhea with antibody titers above the median infected titers, suggesting undetected transmission amongst NHP colony members (Fig. 1D). Time-course analysis revealed stable, elevated IgG titers by one week post detection of infection, whereas IgM responses remained lower throughout the infection, consistent with recall of pre-existing memory responses (fig. S2, F to H).

In general, antibodies against *Shigella* and other bacterial pathogens drive three primary sets of antibacterial functions: 1) complement deposition, 2) Fc receptor engagement, and 3) direct inhibition of virulence (35). Accordingly, we performed three sets of assays with the convalescent serum samples: a serum bactericidal assay (SBA) to measure complement deposition and complement-mediated bacteriolysis, a complement-independent monocyte opsonophagocytosis assay (OPA) to measure Fc receptor engagement and complement-independent phagocytosis, and a HeLa cell-based adhesion and invasion assay (AIA) to measure direct effects on virulence. Convalescent plasma exhibited greater potency relative to asymptomatic controls in all three assays (*P =* 0.0002 for SBA, *P <* 0.0001 for OPA and AIA) (Fig. 1, E to G, and fig. S2, I to K). We next calculated Pearson correlation coefficients for each of the binding and functional serological assays conducted, to evaluate whether any of these immune parameters correlated with symptom severity or with one another. OMVs from the outbreak-associated serotype Y strain were generated in more limited quantities and therefore used only for enzyme-linked immunosorbent assays (ELISAs) with these two weeks post detection of infection samples. Of particular note, IpaD-specific IgM titers were positively correlated with symptom severity (*P =* 0.017), whereas serotype Y OMV IgM titers were inversely correlated (*P =* 0.045) (Fig. 1H). IgM titers against IpaD were also positively correlated with plasma MCP-1 levels (*P =* 0.042), anti-IpaB IgG titers (*P =* 0.001), anti-IpaD IgG titers (*P <* 0.0001), and SBA titers (*P =* 0.028); Anti-Y OMV IgM titers were positively correlated with SBA titers (*P =* 0.003) and OPA titers (*P =* 0.018) (Fig. 1H). These data suggested that antibodies against O-Ag, IpaB, and IpaD may be contributing to the antibacterial immune response. However, these data were purely correlational; they did not demonstrate mechanistically how individual antibodies against specific antigens may be contributing to specific antibacterial functions. Such information is critical for vaccine design, as it can identify antigen epitopes and antibody germline sequences that drive protective immunity (11, 12). We therefore undertook mAb discovery against O-Ag, IpaD, and IpaB.

### Subhead 3: Antibodies against *S. flexneri* O-Ag are functionally diverse

**Figure 2:**
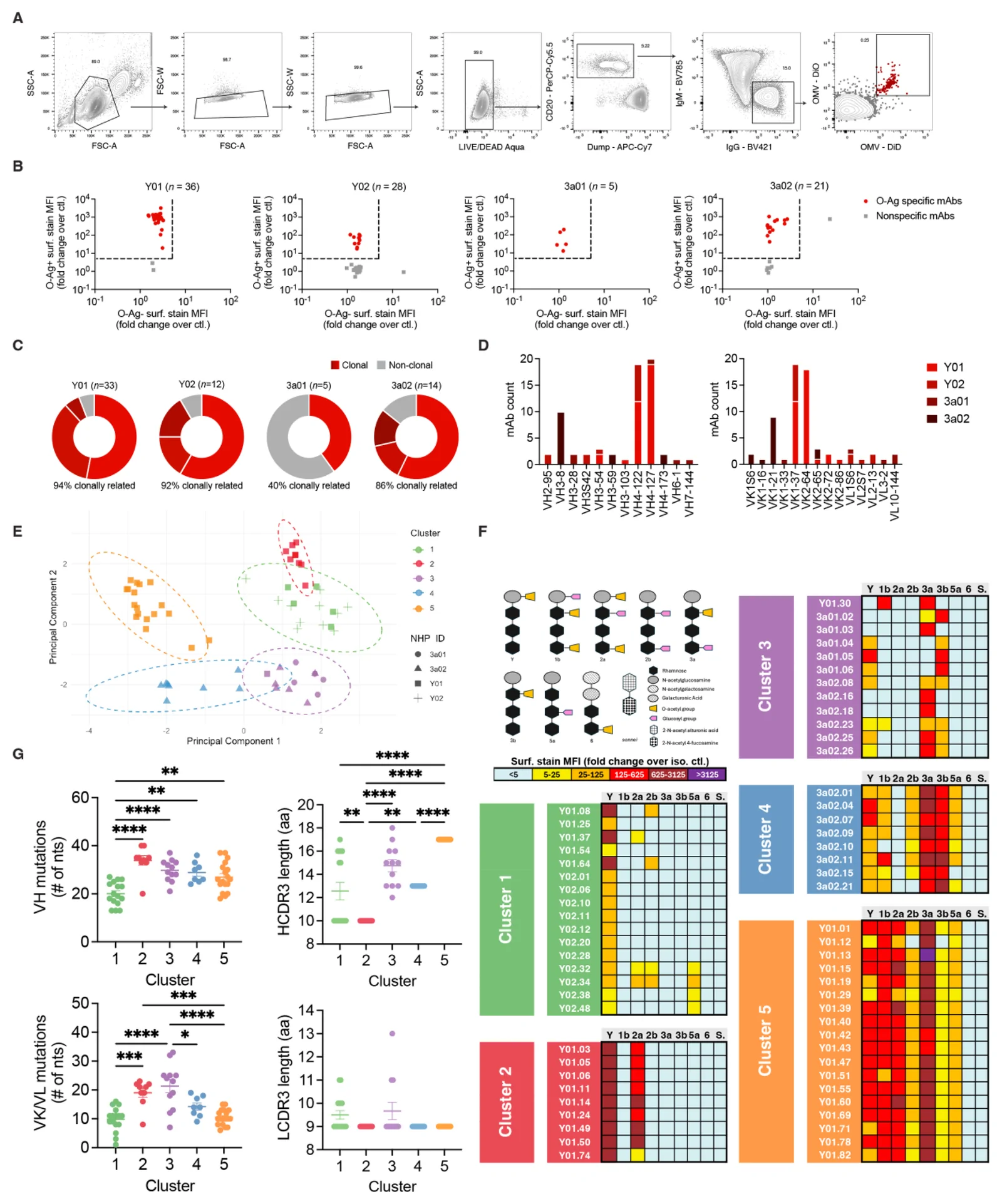
Anti-O-Ag monoclonal antibody profiling revealed variable serotype cross-reactivity. **(A)** Gating strategy for the isolation of OMV-specific memory B cells from NHP PBMCs (LIVE/DEAD^-^, CD3^-^, CD4^-^, CD8^-^, CD14^-^, CD16^-^, CD20^+^, IgM^-^, IgG^+^, OMV^++^). **(B)** O-Ag specific mAbs as determined by fold change over isotype control (ctl.) of surface stain median fluorescence intensity (MFI) for Y/3a O-Ag^+^ and O-Ag^-^ strains. Number of mAbs assayed is shown above each graph. **(C)** O-Ag specific mAb clones (identical VH/VL pairings) for each NHP shown in shades of red; singletons in gray. Total mAbs per NHP indicated above each chart. (**D**) Frequencies of antibody V gene segments per NHP. (**E**) Principal component analysis (PCA) of log-transformed fold change over isotype control of surface stain MFIs against *S. flexneri* serotypes Y, 1b, 2a, 2b, 3a, 3b, 5a, & 6. mAbs are binned by k-means clustering; ellipses show 95% confidence intervals. (**F**) Schematic of O-Ag structures assayed, and heatmap of surface (Surf.) stain values for each mAb across all *S. flexneri* serotypes and *S. sonnei* (S.) compared with isotype control (iso. ctl.). All surface stains were conducted in technical duplicate. (**G**)Mutation frequencies and CDR3 lengths of O-Ag specific heavy chains (upper) and light chains (lower) by PCA cluster (n=8–18 mAbs per cluster). Data were analyzed by ordinary one-way ANOVAs with Tukey’s multiple comparisons tests. *P < 0.05, **P < 0.01, ***P < 0.001, and ****P < 0.0001.

To discover O-Ag-specific antibodies, cryopreserved PBMCs from four convalescent NHPs (Y01, Y02, 3a01, and 3a02) were stained with memory B cell markers (CD20, IgG) and OMVs from the infecting serotype (Y or 3a) were labeled with the lipophilic dyes DiO and DiD. OMV double-positive memory B cells were sorted into 96-well polymerase chain reaction (PCR) plates and paired native heavy and light chain sequences were recovered and cloned into Macaque IgG1 vectors for mAb production and validation (Fig. 2A). Recombinant antibodies were screened for binding to intact bacteria of serotypes Y and 3a and negatively screened against a *S. flexneri* O-Ag knockout strain (Fig. 2B). To further confirm antigen specificity, we performed a lipopolysaccharide (LPS) gel and immunoblot with a representative antibody, demonstrating binding to O-Ag from serotypes Y and 3a, but not the O-Ag knockout strain, with binding restored against the complemented strain (fig. S3, A and B). Across the four OMV sorts, we isolated 64 O-Ag-specific antibodies.

Within most NHPs, we observed a high proportion of clonally related sequences, consistent with prior reports that antibody responses against glycan targets are typically highly clonal (Fig. 2C) (36, 37). Most germline gene pairings were unique to individual NHPs; however, VH4-122 and VK1-37 were shared between animals, comprising 36% of antibodies from Y01 and 58% from Y02 (Fig. 2D). Altogether, the anti-O-Ag mAbs have on average 27 VH nucleotide substitutions (9.5%) and 14 VK/VL nucleotide substitutions (4.9%) (fig. S3C), which is substantial for anti-polysaccharide antibodies (38–40). Furthermore, these mAbs have diverse complementarity determining region (CDR) 3 lengths: 10 to 18 amino acids for heavy chain CDR3s (HCDR3s) and 8 to 13 amino acids for light chain CDR3s (LCDR3s) (fig. S3C). These features suggest that the antibodies were derived from a mature memory response. Though we cannot formally determine how many *Shigella* exposures were required to generate such mAbs, the low serum IgM/IgG ratios observed (fig. S2H) and the evidence presented above for instances of asymptomatic exposure (Fig. 1D) make it probable that such mAb responses were shaped by repeated *Shigella* exposures, not all of which resulted in symptomatic illness. Together, these findings demonstrate that *S. flexneri* infection in NHPs elicits O-Ag-specific B cell responses characterized by substantial somatic hypermutation, despite O-Ag being a T cell independent antigen.

O-Ag responses are generally considered to be serotype-specific, yet polyclonal sera sometimes exhibit cross-serotype reactivity (41, 42). It is unclear whether this breadth arises from a mixture of strictly serotype-specific antibodies, or from the activity of cross-reactive O-Ag antibodies. To assess the serotype specificity of our O-Ag mAbs, we tested each antibody for binding to the surface of *S. flexneri* serotypes Y, 1b, 2a, 2b, 3a, 3b, 5a, 6, and *S. sonnei* (fig. S3D). Principal component analysis of surface stain values grouped antibodies into five distinct functional clusters (Fig. 2, E and F). Cluster 1 comprised primarily Y-specific antibodies from NHPs Y01 and Y02 with limited cross-reactivity to 2a, 2b, and 5a. Cluster 2 consisted of Y- and 2a-cross reactive antibodies from Y01. Cluster 3 contained antibodies from Y01, 3a01, and 3a02, with primary specificity for 3a or 3b and minor reactivity to Y and 1b. Cluster 4 included relatively cross-reactive antibodies from 3a02 that bound Y, 1b, 2b, 3a, 3b, and 5a. Finally, cluster 5 contained the most broadly cross-reactive antibodies, isolated from Y01, which bound all tested *S. flexneri* serotypes except 6. None of the antibodies bound *S. flexneri* 6 nor *S. sonnei*, both of which have distinct O-Ag backbones (Fig. 2F).

Sequence analysis revealed that cluster 1 antibodies, the least cross-reactive, displayed the lowest level of somatic hypermutation and some of the shortest CDR3s (Fig. 2G). In contrast, antibodies from the more cross-reactive clusters tended to exhibit higher levels of somatic hypermutation and longer CDR3s, suggesting that broad cross-reactivity emerges only after extensive affinity maturation (Fig. 2G). Taken together, these data demonstrate that anti-*Shigella* O-Ag antibodies span a spectrum of specificities. Antibodies in our library with a limited number of somatic mutations were more serotype-specific, although those exhibiting a greater degree of maturation could bind multiple *S. flexneri* serotypes.

**Figure 3:**
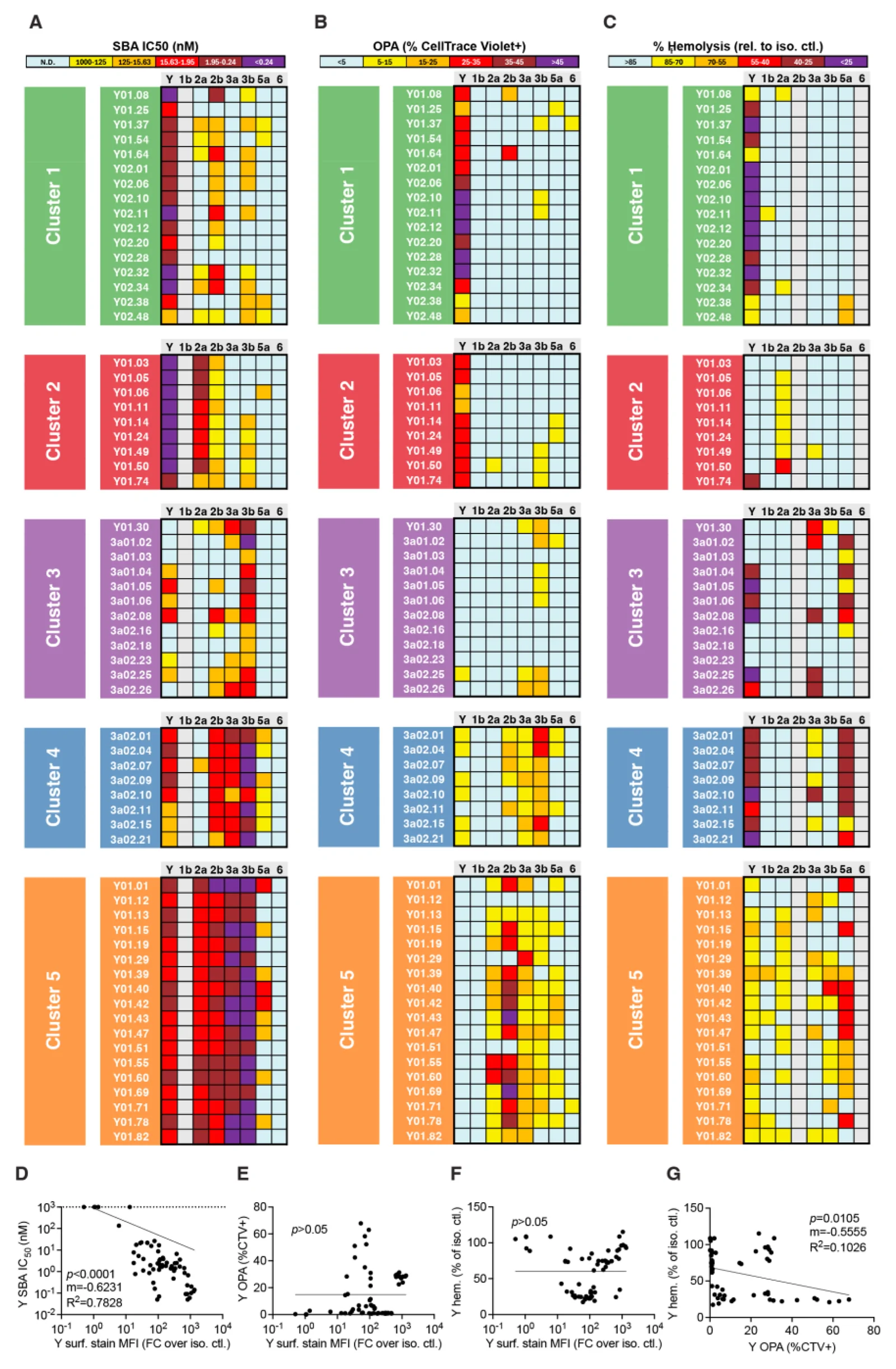
Anti-O-Ag antibody functional analysis identified diverse effector functions. (**A**) SBA IC_50_ values of each O-Ag specific mAb against *S. flexneri* serotypes Y, 2a, 2b, 3a, 3b, 5a, & 6. The serotype 1b isolate used here was too serum sensitive to be used in SBA. All experiments were conducted in technical duplicate. N.D. not detected. (**B**) OPA activity of each mAb against serotype Y, 1b, 2a, 2b, 3a, 3b, 5a, & 6. All experiments were conducted in technical duplicate. (**C**) Percent hemolysis relative (rel.) to isotype control for each mAb against serotypes Y, 1b, 2a, 3a, 3b, & 5a. Our serotype 2b and 6 strains had no detectable hemolytic activity. All experiments were conducted in technical duplicate. (**D to G**) Correlations between serotype Y surface stain and SBA IC_50_ values (**D**), surface stain and OPA activity (**E**), surface stain and hemolysis activity (**F**), and OPA activity and hemolysis activity (**G**). Each data point represents a mAb. mAbs that had no detectable SBA activity were assigned an SBA IC_50_ value of 1,000 nM. For (**D**), data were analyzed with a log-log best fit line. For (**E and F**), data were analyzed with semi-log best fit lines. For (**G**), data were analyzed by simple linear regression. All data were analyzed with extra sum-of-squares F Tests to determine whether the slopes significantly differ from 0.

We next assessed effector functions of our O-Ag specific mAbs across our *S. flexneri* panel, using surface staining and the same functional assays performed above for the serum samples (Fig. 3, A to C). Surface binding was tightly correlated with the ability to coordinate complement-mediated killing (Fig. 3D, and fig. S4A). In contrast to SBA, antibody binding was not as strongly correlated with complement-independent opsonophagocytosis or T3SS inhibition (Fig. 3, E, and F, and fig. S4, B and C). Although OPA and hemolysis assays correlated with surface binding across most strains, they did not against serotype Y, and there were numerous exceptions in which mAbs bound the bacterial surface of a particular strain but did not have activity in OPA or hemolysis assays (Fig. 3, E and F, and fig. S4, B and C). Furthermore, opsonophagocytic activity and T3SS inhibition were only weakly correlated with each other, and there were numerous instances in which mAbs coordinated one function but not the other (Fig. 3G and fig. S4D). Taken together, these results indicate that, although surface binding of anti-O-Ag antibodies is generally sufficient to trigger complement-mediated bacteriolysis among *S. flexneri* strains, it is not sufficient to trigger complement-independent opsonophagocytosis and T3SS virulence inhibition. These functions may be more sensitive to factors beyond O-Ag binding, such as the distinct geometry or kinetics of antibody binding.

### Subhead 4: IpaD and IpaB elicit T cell responses in infected macaques

Antibodies are important components of acquired immunity against *Shigella* infection (43), but T cells also play an important role (44). As a polysaccharide, O-Ag cannot induce major histocompatibility complex (MHC)-dependent T cell activation; in contrast, the protein antigens IpaD and IpaB have been directly shown to elicit T cell responses in mice (45). Therefore, we investigated whether they elicited T cell responses in our NHP samples. PBMCs were stimulated in vitro with IpaD, IpaB, or the positive control superantigen Staphylococcus enterotoxin B (SEB) (46), and activation was assayed by concentrations of T helper cytokines in media and by flow cytometry analysis of both surface activation markers and intracellular cytokines (fig. S5A). Cytokine profiling of supernatants showed induction of IL-21, IL-10, IL-6, and tumor necrosis factor (TNF)-α (all *P <* 0.0001), and no significant (all *P >* 0.05) effect on interferon (IFN)-γ, interleukin (IL)-2, IL-13, IL-4, IL-5, or IL-17A (fig. S5B). Flow cytometry revealed activation of CD4 and CD8 T cells by IpaD and IpaB, measured by the activation induced markers (AIM) OX40 and 4-1BB (fig. S5, C and D). Intracellular staining confirmed induction of IFN-γ in CD8 T cells, NK cells, and NKT cells, and induction of IL-17A in CD8 T cells and NK cells, but no induction of either cytokine in CD4 T cells (fig. S5, E and F). These results indicate that both IpaD and IpaB elicit cellular immune responses in NHPs, though without broad effects on IFN-γ and IL-17A expected based on previous reports (44, 45).

### Subhead 5: Antibodies against the T3SS can both inhibit and enhance virulence

**Figure 4:**
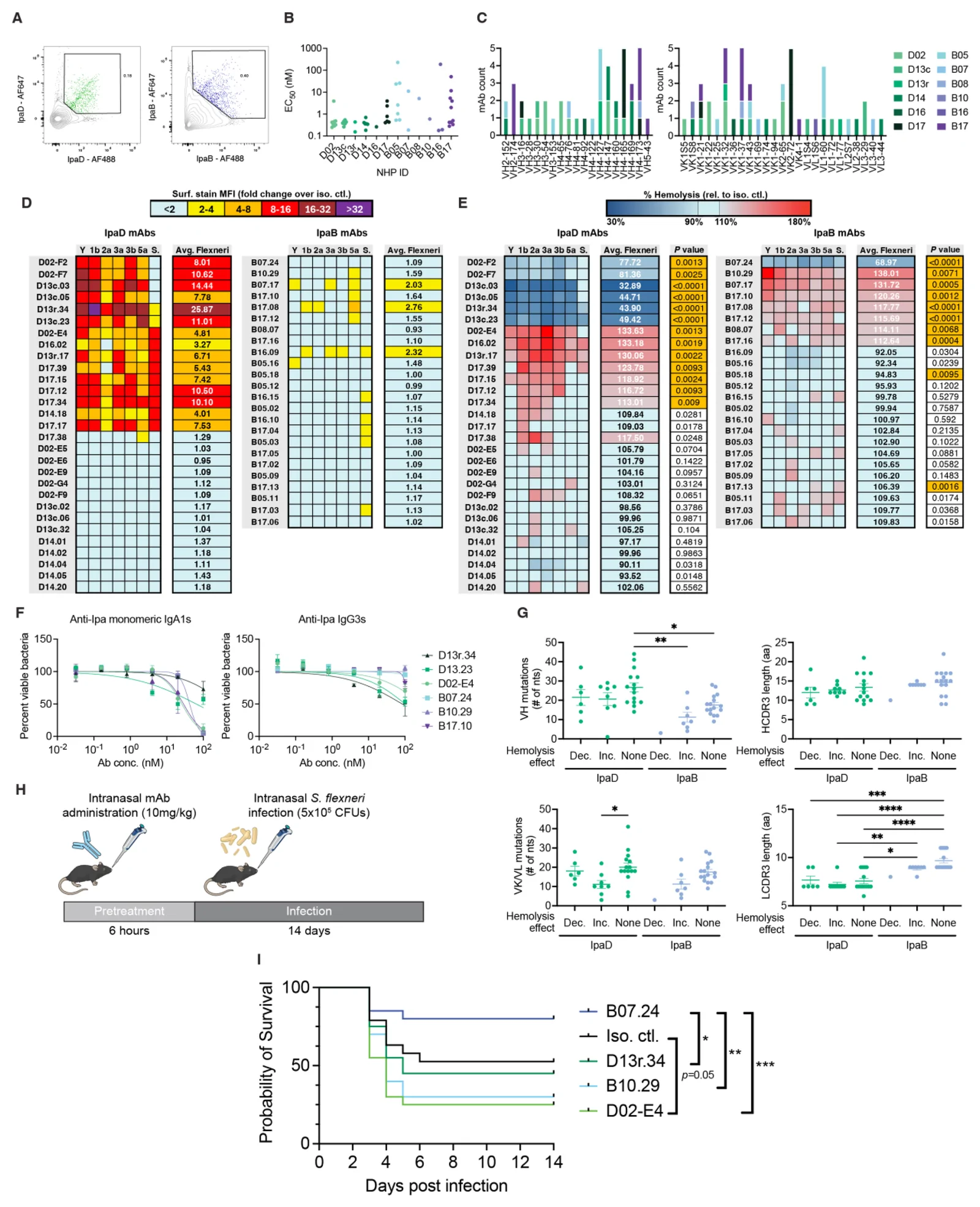
Anti-T3SS antibody characterization demonstrated both inhibitory and enhancing effects on T3SS activity. (**A**) Gating strategy for isolation of Ipa-specific memory B cells (LIVE/DEAD^-^, CD3^-^, CD4^-^, CD8^-^, CD14^-^, CD16^-^, CD20^+^, IgM^-^, IgG^+^, Ag^++^) from NHP PBMCs. (**B**) ELISA antigen EC_50_ values of Ipa-specific mAbs. (**C**) Frequencies of antibody V gene segments identified for each NHP. (**D**) Surface stain MFI fold change over isotype control for each Ipa-specific mAb against *S. flexneri* serotypes Y, 1b, 2a, 3a, 3b, & 5a, and against *S. sonnei* (S.). Bacteria were grown in the presence of 0.1% DOC for IpaB surface stains. (**E**) Percent hemolysis relative to isotype control of each Ipa-specific mAb against the same panel as (**D**). Data were analyzed by one sample t tests of combined *flexneri* hemolysis, compared to 100% hemolysis relative to isotype control. *P* < 0.01 is considered significant. (**F**) SBA curves of representative Ipa-specific mAbs in IgA1 monomer and IgG3 formats. Experiments were conducted in technical duplicate three times, with one representative experiment shown. (**G**) Mutation frequencies and CDR3 lengths of Ipa-specific mAbs, grouped by hemolysis effects (Dec.: decreases hemolysis, Inc.: increases hemolysis, None: no effect). (n=1–15 mAbs per group) Data were analyzed by ordinary one-way ANOVAs with Tukey’s multiple comparisons tests. (**H**) Schematic of in vivo experiment. C57BL/6J mice were intranasally administered 10 mg/kg of mAb, then infected 6 h later with 5 × 10^5^ CFU *S. flexneri* 2a (2457T). Mice were monitored daily for 14 days. (**I**) Survival of mice pretreated with each mAb. n = 20 per condition, two independent experiments combined. Data were analyzed by log-rank analysis. *P < 0.05, **P < 0.01, ***P < 0.001.

We next examined antibody responses against IpaD and IpaB. Memory B cells were identified from cryopreserved PBMCs from six convalescent NHPs for IpaD (D02, D13c, D13r, D14, D16, and D17) and six for IpaB (B05, B07, B08, B10, B16, and B17) using the same gating strategy as our O-Ag specific antibody discovery (Fig. 2A). IpaD-specific B cells were identified using biotinylated monomeric IpaD complexed with AF488 and AF647-conjugated streptavidin as sorting baits, and IpaB-specific B cells were identified using IpaB labeled with AF488 and AF647-conjugated NHS-esters (Fig. 4A). Antigen double-positive memory B cells were sorted into 96-well plates and paired heavy and light chain sequences were recovered (Fig. 4A). Antibodies were screened by ELISA for antigen binding, and counter-screened against ovalbumin as a nonspecific control (fig. S6, A and B). Across the twelve Ipa protein sorts, we isolated 29 IpaD-specific antibodies and 24 IpaB-specific antibodies. Nearly all IpaD-specific antibodies were high affinity: 26 antibodies exhibited sub-nanomolar half-maximal effective concentration (EC_50_) values by ELISA, and the remaining three had EC_50_ values below 10 nM (Fig. 4B). The IpaB antibodies exhibited a wide range of affinities: 11 were high affinity with sub-nanomolar ELISA EC_50_ values, but the remainder had a wide range of affinities as low as 228 nM (Fig. 4B). Both anti-IpaD and anti-IpaB antibodies were derived from diverse germline gene pairings. VH4-173 and VK1-37 were paired in both B08 and B17, but all other germline pairings were unique to each NHP (Fig. 4C). Sequence analysis revealed comparable somatic hypermutation between the two libraries, averaging 24 VH nucleotide substitutions (8.1%) and 17 VK/VL nucleotide substitutions (6.0%) for IpaD-specific antibodies, and 21 VH nucleotide substitutions (7.3%) and 15 VK/VL nucleotide substitutions (5.3%) for IpaB-specific antibodies (fig. S6C).

The T3SS needle tip consists of a conformationally dynamic complex including pentameric IpaD (47) and (most likely) monomeric IpaB (48). Because all our mAbs were isolated using soluble, monomeric, recombinant protein, we assessed binding of these mAbs to native surface-exposed IpaD and IpaB on *S. flexneri* strains Y, 1b, 2a, 3a, 3b, and 5a (fig. S6D). Fifteen antibodies bound to surface IpaD on at least five strains, with highly consistent binding patterns across serotypes (Fig. 4D). In contrast, even with a modified surface stain protocol performed in the presence of deoxycholate (a bile salt known to recruit IpaB to the needle tip (55)) and amplified binding signal using a polyclonal tertiary antibody, IpaB-specific antibodies exhibited weak and variable surface staining (Fig. 4D). *S. sonnei* shares 97.3% IpaD and 99.0% IpaB sequence identities with *S. flexneri*, and both antigens have previously been shown to elicit cross-protective immune responses (45). Therefore, we also tested our antibody libraries for binding to *S. sonnei*. Eleven of the 15 *S. flexneri* IpaD binding antibodies also bound *S. sonnei*, whereas the IpaB antibody library exhibited similarly weak and inconsistent binding to *S. sonnei* (Fig. 4D).

Functional assays revealed that IpaD-specific antibodies lacking surface binding had no impact on T3SS function (Fig. 4E). Nearly all surface-binding IpaD antibodies impacted hemolysis, but much of the impact appeared harmful: six blocked hemolysis, but seven enhanced it (Fig. 4E). Four of the blockers and five of the enhancers had no effect on S. sonnei, demonstrating that cross-species functional activity is limited at the monoclonal level (Fig. 4E). Despite the inconsistent IpaB surface staining, hemolysis assays revealed consistent effects across both *S. flexneri* and *S. sonnei*: two IpaB-specific antibodies reduced hemolysis, eight enhanced it, and the remainder had no effect (Fig. 4E). Consistent with these results, commercially available rabbit anti-IpaB polyclonal antibodies enhanced *Shigella*-dependent hemolysis of red blood cells in vitro (*P <* 0.0001) (fig. S6E).

Neither anti-IpaD nor anti-IpaB IgG1 antibodies mediated SBA or OPA against *S. flexneri* serotype Y (fig. S6, F and G). Given that these functions are isotype-dependent, we re-cloned a subset of representative antibodies into IgG3 and monomeric IgA1 backbones. IgA1 has been shown to activate complement through non-classical routes in some contexts, and IgG3 is well established to engage C1q efficiently (49, 50). Isotype-switched antibodies did not exhibit OPA activity (fig. S6H), but some did weakly coordinate complement-mediated killing (Fig. 4F).

Sequence analysis revealed no major differences in somatic hypermutation nor CDR3 lengths between antibodies with distinct functional profiles within each antigen-specific library, suggesting that antibody-mediated functional modulation of T3SS activity does not require unusually complex maturation pathways (Fig. 4G). IpaD antibodies tended to have more VH mutations whereas IpaB antibodies tended to have longer LCDR3s (Fig. 4G). Collectively, these findings indicate that IpaD and IpaB responses in NHPs are polyclonal and diverse but consistently affinity matured. Also, they can modulate T3SS activity across serotypes and can weakly coordinate complement-mediated killing as IgG3 and monomeric IgA1 antibodies.

One other anti-*Shigella* T3SS mAb, the anti-IpaD mouse antibody IpaD-318, has been evaluated in vivo, using a murine intranasal challenge model (51). IpaD-318 enhanced hemolysis in vitro, but paradoxically protected mice from *Shigella* challenge in vivo. Given this context, we next tested whether our infection-derived antibodies were sufficient to impact in vivo disease, and whether enhancers and blockers were different from one another. Five antibodies, including two hemolysis blockers (D13r.34 & B07.24), two hemolysis enhancers (D02-E4 & B10.29), and one isotype control (VRC01, an antibody against human immunodeficiency virus GP120 (52)), were administered intranasally (10 mg/kg) to C57BL/6J mice 6 hours prior to a challenge with a half-maximal lethal dose [LD_50_; 5 × 10^5^ colony-forming units (CFUs)] of *S. flexneri* 2a 2457T (Fig. 4H). We elected to use 2457T for this challenge study since it has been used extensively to profile IpaB and IpaD-mediated immunity (45, 51), and our hemolysis assays confirm these mAbs influence its T3SS function (Fig. 4E). We observed different rates of survival across mice treated with different mAbs, with mice treated with the blocker B07.24 exhibiting 75% survival, mice treated with the blocker D13r.34 and the isotype control exhibiting 45-55% survival, and mice treated with the enhancers D02-E4 and B10.29 exhibiting 25-30% survival (Fig. 4I). Mice treated with B07.24 had significantly increased survival relative to the enhancers B10.29 (*P <* 0.01), and D02-E4 (*P <* 0.001), as well as the blocker D13r.34 (*P <* 0.05), indicating that hemolysis blockers and enhancers do not function identically in vivo (Fig. 4I and fig. S6I). None of observed differences in survival between isotype control treated animals and those treated with B07.24 (*P* = 0.0824), B10.29 (*P* = 0.1555), or D02-E4 (*P* = 0.0545) were statistically significant. Thus, anti-IpaD and anti-IpaB mAbs that either block or enhance hemolysis in vitro appear have differential effects on disease relative to each other in vivo, and the direction of this difference is generally consistent with the direction of their in vitro effect.

### Subhead 6: Anti-IpaD and anti-IpaB antibody functions map to specific epitopes

**Figure 5:**
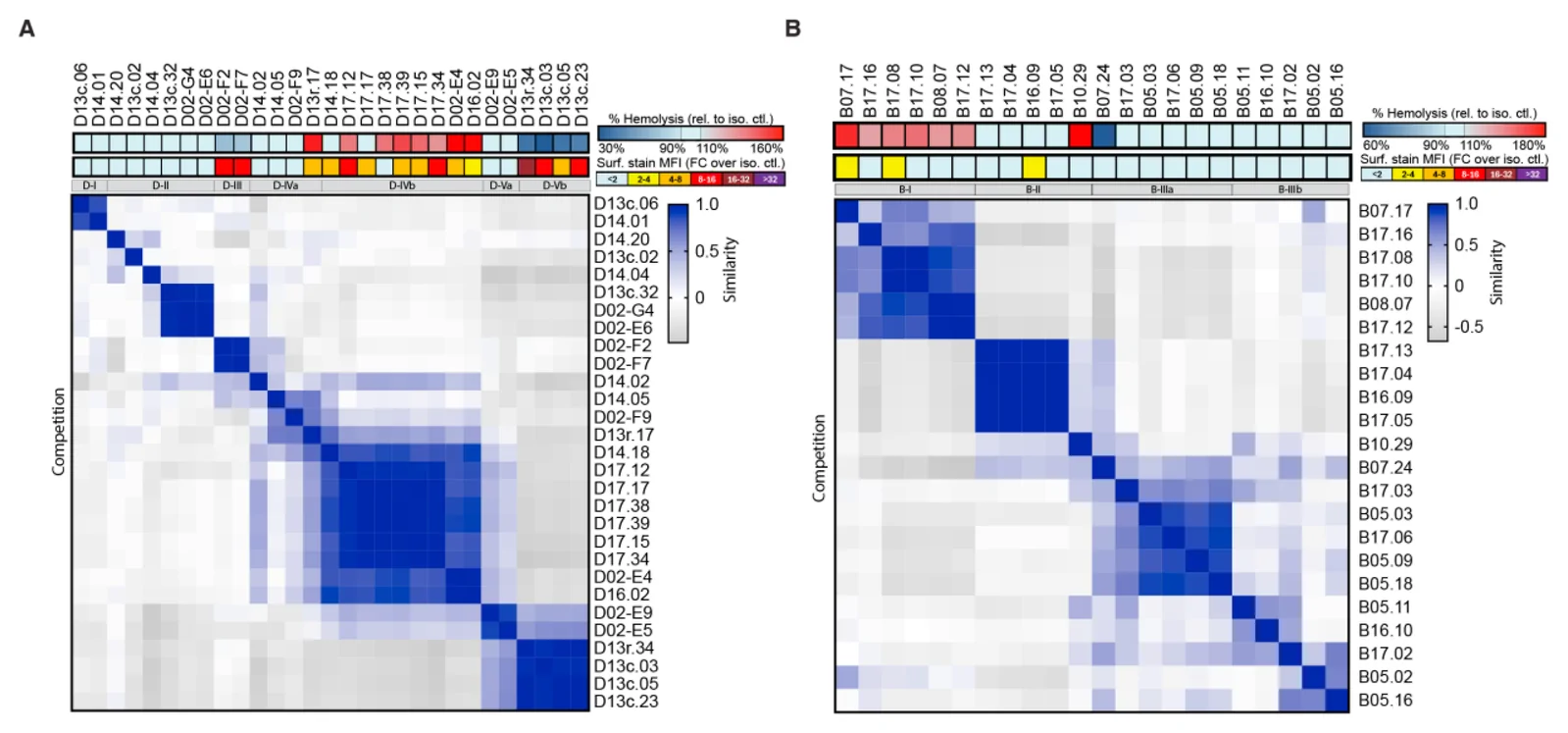
Epitope binning revealed anti-T3SS antibodies cluster into functionally distinct epitopes. (**A and B**) Competition ELISA of IpaD-specific mAbs (**A**) and IpaB-specific mAbs (**B**). Values were normalized to unblocked and self-blocking controls. FC, fold change. Experiments were conducted in technical duplicate. A Pearson-correlation similarity matrix was applied. Average surface stain and hemolysis values of each mAb relative to isotype control across all *flexneri* serotypes assayed are indicated above.

Antibodies against a given target may have opposite functional effects either because they bind a different epitope or because they trigger different conformational changes when binding to the same epitope. Therefore, given the distinct functional effects observed for the mAbs against both IpaD and IpaB, we next examined whether these differences may reflect differences in epitope specificity. Competition ELISAs revealed that functional IpaD antibodies clustered into two hemolysis-blocking epitopes (clusters D-III and D-Vb) and one enhancing epitope (cluster D-IVb) (Fig. 5A). Most IpaB hemolysis-enhancing antibodies clustered into one dominant epitope (epitope B-I) (Fig. 5B). The most potent mAbs B10.29 (the strongest in vitro hemolysis enhancer) and B07.24 (the strongest in vitro hemolysis blocker) both spanned two otherwise non-functional epitopes (clusters B-II and B-IIIa) (Fig. 5B).

**Figure 6:**
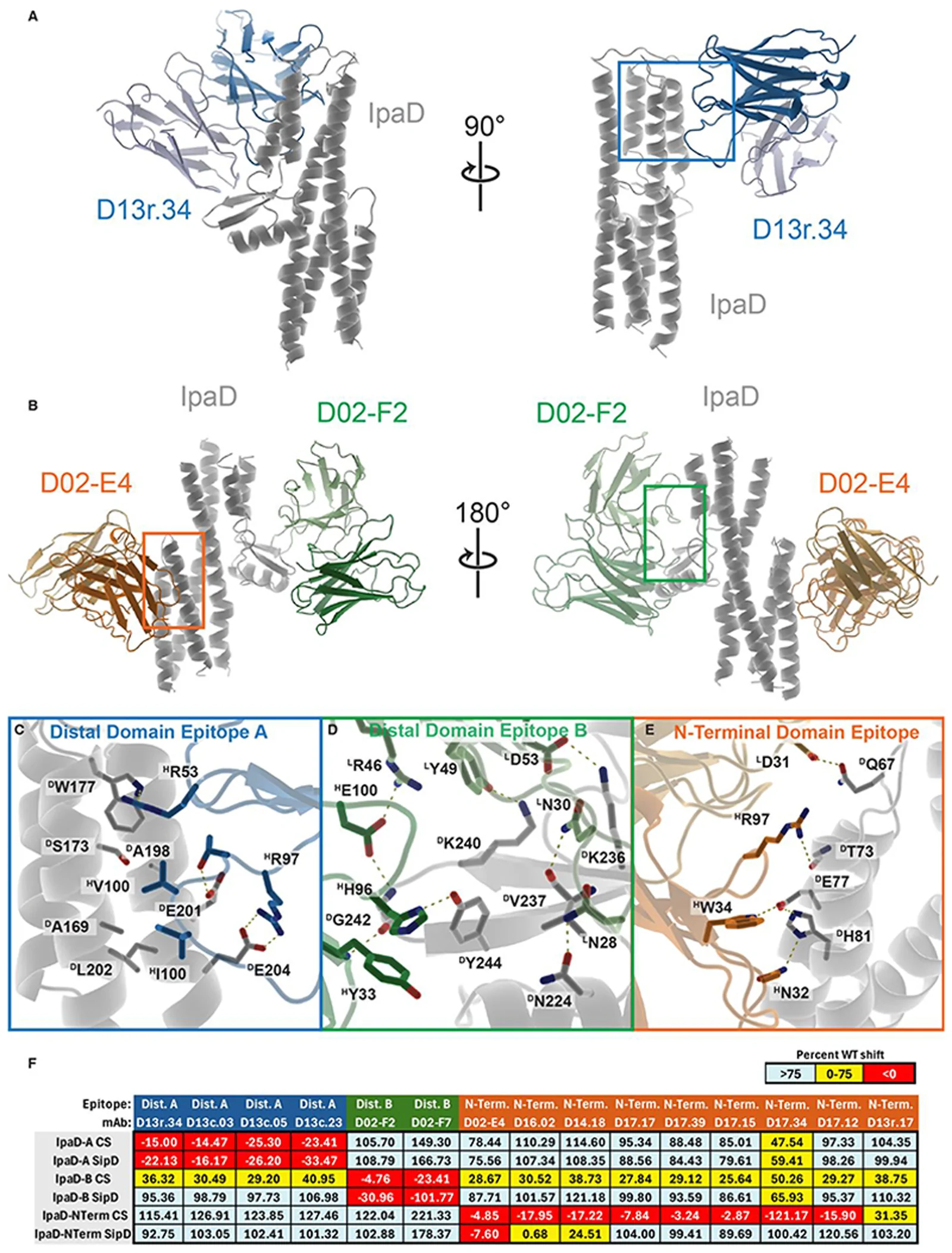
Structural analysis demonstrated distinct IpaD domains are targeted by hemolysis-enhancing and -blocking antibodies. **(A)** Cryo-EM structure of IpaD (gray) in complex with D13r.34 (blue). The mAb light chain is depicted in a lighter color. The blue box shows the region depicted more closely in (C). **(B)** Cryo-EM structure of IpaD (gray) in complex with D02-F2 (green) and D02-E4 (orange). The mAb light chains are depicted in a lighter color, and the green and orange boxes show regions depicted more closely in (D) and (E), respectively. (**C to E**) Depictions of the Fab-IpaD interface for D13r.34 (blue) (**C**), D02-F2 (green) (**D**), and D02-E4 (orange) (**E**), showing key interactions. Residues are prefaced with a “D” if they are part of IpaD, “H” if they belong to the mAb heavy chain, and “L” if they belong to the mAb light chain. Likely electrostatic interactions are shown as yellow dashed lines. Light chains are depicted in a lighter shade than heavy chains, and IpaD is gray. (**F**) Biolayer interferometry analysis showing relative binding of each antibody to mutagenized IpaD constructs. Data are expressed as percent binding signal (shift) normalized to wildtype (WT) IpaD shift for each antibody.

To identify these functional epitopes on both IpaD and IpaB, we sought to determine representative mAb:antigen complex structures by cryogenic electron microscopy (cryo-EM). The structure of full-length IpaB has not yet been solved and our attempts at IpaB structural determination were unsuccessful. However, we did determine that B10.29 was the only antibody capable of binding in an ELISA to IpaB28.226, a domain essential for IpaB-IpaD binding (53) (fig. S7A). IpaD has a well-defined structure with eight α-helices and five β-strands arranged in three domains: the N-terminal domain, the central coiled-coil domain, and the distal domain (54) (fig. S7B). Complexes between IpaD and two representative hemolysis blocking mAbs (D02-F2 and D13r.34) and one hemolysis enhancing mAb (D02-E4) were resolved in two different structures: one with IpaD in complex with D13r.34, and another with IpaD in complex with both D02-F2 and D02-E4 (Fig. 6, A and B, fig. S8, fig. S9, and table S1).

Our structures identified mAb binding sites at the domain level. D13r.34 bound α3 of the central coiled-coil domain and α4 of the distal domain in a manner that appeared to be primarily dependent on its CDRH3 (Fig. 6, A and C, and fig. S10, A and B). This epitope, which we call distal domain epitope A, is nearly identical to those previously reported for JPS-G3, JMK-E3, and 20ipaD, camelid VHH domains that inhibit *Shigella* hemolysis (fig. S10C) (54). In contrast, D02-F2 and D02-E4 bound regions of IpaD not recognized by previously reported IpaD mAbs (51, 54). Both the heavy and light chains of D02-F2 were found to be in close contact with what we call distal domain epitope B, the distal domain at βC, βD, α5, and βE (Fig. 6, B and D). In contrast, the hemolysis enhancing mAb D02-E4 associated closely with what we call the N-terminal domain epitope, at α1 and α2, again with both heavy and light chains likely playing roles in IpaD recognition (Fig. 6, B and E). One additional protective IpaD epitope has previously been described in the literature, and it is distinct from those identified in this study (fig. S10D) (58).

To validate these epitopes and lay a foundation for rational antigen design, we introduced targeted mutations within distal domain epitope A, distal domain epitope B, and the N-terminal domain epitope. The mutations were either conservative substitutions with homologous Salmonella SipD residues, or more disruptive charge-swap (CS) mutations (fig. S11A). Binding to wildtype IpaD was detectable for all antibodies using biolayer interferometry (BLI) (fig. S11B). Both IpaD-A variants (SipD and CS) abolished epitope-specific binding (Fig. 6F and fig. S11C). The IpaD-B SipD variant abolished epitope-specific binding, whereas the IpaD-B CS variant disrupted all antibody binding (Fig. 6E, and fig. S11D). The IpaD-NTerm SipD variant only ablated D02-E4 binding, whereas the IpaD-NTerm CS variant also blocked binding of other antibodies targeting that site (Fig. 6E, and fig. S11E). Altogether, these data demonstrate that functional differences among anti-Ipa protein mAbs are consequences of distinct binding epitopes, and our preliminary epitope-specific IpaD mutagenesis suggests that rational design of IpaD variants may enable selective ablation of disease-enhancing antibody responses while preserving those that lead to inhibition of hemolysis.

## Discussion

Previous work in NHP challenge models (13) and in humans (16, 17) suggested that acquired immunity to *Shigella* infection is limited in breadth (often being serotype-specific) and duration. Systems-level analysis of clinical samples suggests that what protection is acquired is correlated with titers against candidate vaccine antigens that include O-Ag (55) and T3SS proteins (19, 20, 56). Such data have guided the current vaccine pipeline (5); however these studies necessarily provide only correlative associations between immunological metrics and disease without sufficient resolution or mechanistic insight to direct the rational design of vaccine antigens. Here, we leveraged a natural outbreak of circulating *S. flexneri* strains in an NHP research colony to dissect antibody responses against candidate vaccine antigens (O-Ag, IpaB, and IpaD), with the aim of better understanding how to design effective *Shigella* vaccines.

The key finding from this anti-O-Ag mAb discovery campaign was that a subset of these mAbs have sufficient breadth to bind to and coordinate complement-mediated bacteriolysis against many *S. flexneri* serotypes (Fig. 2F and Fig. 3A). Although O-Ag is the most common antigen in candidate *Shigella* vaccines due to evidence for its importance in infection (5, 13–17), its high structural variability between serotypes (57) makes achieving clinically relevant breadth challenging (5). Immunization with OMVs from as few as four *Shigella* strains can provide broad, O-Ag-dependent serum bactericidal responses in rats and rabbits, though not humans (58). An open question is whether broad and potent O-Ag responses consist primarily of many unique antibody lineages specific for a narrow range of serotypes, or a few lineages that are cross-reactive. Here we find clonally-related, broadly-reactive mAbs that have undergone extensive (for a T cell-independent antigen) affinity maturation (Fig. 2G). These data imply it is possible to produce such mAbs, and the high degree of somatic hypermutation observed leads us to hypothesize that these mAbs arise following repeated *Shigella* infections. Indeed, the low clonal diversity and relatively high number of variable domain mutations in broad mAbs (Fig. 2G) suggest that such antibodies may be challenging to elicit. One other study has reported comparable levels of somatic hypermutation in anti-O-Ag mAbs (against *Klebsiella pneumoniae*) (59). A recent analysis of mAbs isolated from humans immunized with a candidate *S. sonnei* vaccine showed levels of somatic hypermutation like those of the serotype-specific cluster 1 mAbs reported here (60), suggesting that alternative vaccine dose schedules or formulations could be required to elicit broad antibodies against O-Ag. Future work involving lineage tracing and characterization of broad anti-O-Ag mAbs will be important to determine how much maturation is required to achieve optimal breadth.

Although most IpaD- and IpaB-specific antibodies we isolated were non-functional, both antigens could elicit mAbs that potently inhibit hemolysis with relatively few mutations in a diverse set of germline genes (Fig. 4, C and G) and induce cellular immunity (fig. S5). In contrast to O-Ag-specific mAbs, T3SS-directed mAbs were more uniformly cross-reactive against *S. flexneri* serotypes, and several exhibited cross-species activity against *S. sonnei*. However, we also observed evidence of in vitro enhancement of virulence by some mAbs (Fig. 4E). Although previous has reported that the anti-IpaD mAb IpaD-318 enhanced virulence in vitro but was protective in vivo (51), we observed that the enhancing mAbs reported here had different effects on survival than the strongest blocking mAb (Fig. 4I) in a murine challenge model, suggesting that the in vitro enhancement of virulence observed is not an assay artifact. It should be noted that the in vivo assay used here was primarily intended to determine whether the effects of the blocking and enhancing mAbs tested were directionally different from one another, and the LD50 challenge approach means that it is difficult to establish statistically significant differences between the mAbs tested and the isotype control since the baseline survival rate is 50% by design. Given this, the in vitro hemolysis data, murine challenge data, and the correlation observed between anti-IpaD IgM titers and disease severity (Fig. 1H) lead us to hypothesize that ADE could be a feature of at least a subset of *Shigella* infections. ADE has been most notoriously associated with viral pathogens such as Dengue virus (61) but has also been reported for some bacterial pathogens (62–66). *Shigella* is already known to employ strategies that suppress innate (67, 68) and T cell responses (69, 70), and if future works confirms that ADE is indeed a feature of some *Shigella* infections, this would be another means that the pathogen could use to subvert the host immune system. Importantly, in vitro protective and virulence-enhancing effects mapped onto distinct epitopes of both IpaB and IpaD (Fig. 5, A and B). The strongest in vitro hemolysis enhancing IpaB-specific mAb (B10.29) bound to the IpaD-binding N-terminal domain, suggesting it may modulate IpaD-IpaB interactions (53). Cryo-EM analysis of IpaD-mAb complexes revealed that in vitro hemolysis-blocking mAbs primarily bind the distal domain, whereas the in vitro hemolysis-enhancing mAbs bind to the N-terminal domain (Fig. 6, A to E). The distal domain of IpaD moves as the T3SS complex is primed for invasion (47, 71), and a structure of the *Salmonella* T3SS needle tip complex shows SipD in a conformation incompatible with the N-terminus position observed in monomeric crystal structures (72). Thus, it is tempting to speculate that within the assembled T3SS needle tip, IpaD’s N-terminal and distal domains sample conformational states that have different capacities for host cell invasion (73), and these conformational states may be stabilized by specific mAbs. By mutating IpaD we ablated antibody binding to each specific epitope (Fig. 6F), suggesting that IpaD can be rationally designed to avoid eliciting antibodies targeting in vitro hemolysis-enhancing epitopes. Future work should evaluate whether these redesigned IpaD constructs retain immunogenicity and elicit protective antibody responses, and whether antibody responses against IpaD distal domain epitopes A and B are protective in vivo. Taken together, these data indicate that Ipa proteins may be rationally designed to specifically elicit protective immunity without ADE of virulence, should this prove to be a phenomenon in some *Shigella* infections.

This study has limitations that need to be considered. First, we were unable to collect samples from any infected animal prior to infection, precluding analysis of correlates of protection. Thus, although we can identify serological features that are correlated with disease severity, we cannot make claims about full protection. Second, we focused our analysis on a few key assays (surface staining, SBA, complement independent OPA, and hemolysis assay/AIA), each chosen to represent a key antibacterial antibody function (whole cell binding, complement deposition/bacteriolysis, Fc receptor engagement/opsonophagocytosis, and virulence inhibition, respectively). However, additional functions not assayed here, such as complement-dependent opsonophagocytosis by macrophages or neutrophils, or NK cell activation, may also be important for protective immunity. Future work should examine the capacity of different antibodies to drive such effector functions against *Shigella*. Third, we were able to isolate mAbs from infected and symptomatic animals that were either protective or pathogenic in vitro and differed from one another in a murine lung challenge model, but we are currently unable to make definitive claims as to whether these specific mAb populations impact disease severity during natural infection. Rather, this work demonstrates that broad anti-O-Ag mAbs, inhibitory T3SS mAbs, and virulence-enhancing mAbs are elicited during natural *Shigella* infection in NHPs, suggesting that selective elicitation of these types of antibodies via rational vaccine design may offer a path towards more effective vaccines.

These limitations notwithstanding, this mAb discovery campaign revealed unexpected features of the anti-*Shigella* antibody response in naturally-infected NHPs and represents a step towards the rational design of *Shigella* vaccine candidates. Future work should prioritize additional structural insights into anti-*Shigella* mAb function and determine if highly matured, broad anti-O-Ag mAbs and disease enhancing mAbs against T3SS antigens arise in humans. More broadly, it will also be important to determine the prevalence of highly matured anti-polysaccharide antibodies and the potential for T3SS-derived ADE among other pathogens. Our findings suggest that integrating structural and immunological data into *Shigella* vaccine design strategies can likely overcome existing challenges to create broad and efficacious vaccines, with implications for bacterial vaccine design more generally.

## Materials and Methods

### Study design

This study assesses the bacterial genomics and host immune response of an NHP *Shigella* outbreak. Bacterial isolates were analyzed from all infections during the study period, blood draws for immunological analyses were collected from any NHPs not assigned to a study at the time of diagnosis. Positive and negative controls were included in all in vitro experiments. When these controls yielded the expected results (negative control antibody does not differ from buffer only control, and positive control antibody demonstrates the statistically significant effect we expect compared to negative control), we included all samples from that experiment in analyses. All in vitro experiments were conducted in duplicate and pooled independent experiments are indicated in figure legends. Experimenters were not blinded for in vitro assays. Mice were commercially obtained and randomly assigned to experimental groups. Group sizes were based on a two-sided power analysis for comparison with an LD_50_ control group using α = 0.05; with 20 mice per group total across two independent experiments, the study was powered to detect at least a 30% shift in mortality (more than 80% or less than 20% mortality in treated groups). The experimenter was blinded to conditions for mouse survival studies.

### Statistical analysis

Individual-level data for experiments with *n* < 20 are presented in data file S1. Statistical tests were performed using Prism 10.6.1, and the sample sizes, statistical tests used, and *P* values for each figure and panel are indicated in the figure legends. Normality and log-normality were determined using the Shapiro-Wilk test, and IC_50_ and EC_50_ values were calculated by fitting data with four-parameter logistic curves. Comparisons of IgG and IgM titers between infected and asymptomatic animals (Fig. 1D) were performed using multiple lognormal Welch’s *t* tests with Holm-Šídák correction for multiple comparisons; the same approach was used to compare plasma cytokine concentrations between asymptomatic and infected animals (fig. S2D). Functional serological assays (Fig. 1, E to G) were analyzed by a lognormal Welch’s *t* test (SBA) or Welch’s *t* tests (OPA and AIA), and the polyclonal anti-IpaB hemolysis comparison (fig. S6E) was analyzed by an unpaired *t* test with Welch’s correction. Differences in symptom index by sex (fig. S2B) were assessed by an unpaired *t* test with Welch’s correction, and the relationship between symptom index and age (fig. S2C) was assessed by simple linear regression. Relationships among symptom severity, plasma cytokines, antibody titers, and functional titers (Fig. 1H), as well as epitope-binning similarity (Fig. 5), were evaluated using Pearson correlation coefficients. Surface-staining profiles of O-Ag-specific mAbs across *S. flexneri* serotypes (Fig. 2, E and F) were grouped by principal component analysis with *k*-means clustering. Differences in somatic hypermutation frequencies and CDR3 lengths across antibody clusters, functional groups, or individual NHPs (Fig. 2G, Fig. 4G, and figs. S3 and S6) were assessed by ordinary one-way ANOVA with Tukey’s multiple comparisons test. Correlations between surface binding and effector functions, both overall and by serotype (Fig. 3, D to G, and fig. S4), were evaluated using an extra sum-of-squares *F* test to determine whether the best-fit slope differed significantly from zero. T cell activation readouts, including secreted cytokine concentrations and activation-induced marker expression following PBMC stimulation (fig. S5), were analyzed by ordinary one-way ANOVA with Dunnett’s multiple comparisons test. Hemolysis activity of individual monoclonal antibodies (Fig. 4E) was assessed by one-sample *t* tests comparing combined *S. flexneri* hemolysis to 100% hemolysis relative to the isotype control, with *P <* 0.01 considered significant. Differences in survival among mice treated with different monoclonal antibodies (Fig. 4I) were evaluated by log-rank analysis. Unless otherwise noted, *P <* 0.05 was considered statistically significant (*P < 0.05, **P < 0.01, ***P < 0.001, ****P < 0.0001).

## Supplementary Material

Data File S1

Supplemental Material

## Data Availability

All data associated with this study are in the paper or supplementary materials. Bacterial isolate WGS reads have been deposited to NCBI under BioProject PRJNA1391884 with BioSample IDs SAMN54236081 to SAMN54236176. Antibody variable region sequences have been deposited to NCBI with GenBank accession numbers PX758762 to PX758997. Structural data have been deposited to the Protein Database (PDB) with accession numbers 9ZRT (IpaD– D13r.34) and 9ZRS (IpaD– D02-F2– D02-E4). All materials used or generated in this study are commercially available or will be supplied upon reasonable request.

## Editor’s Summary

Understanding an Outbreak. Understanding the response elicited to an infection can inform vaccine and therapeutic design strategies. Gallant *et al*. leveraged an outbreak of *Shigella flexneri* in a nonhuman primate facility with this goal in mind. The authors found that antibodies were elicited against the O-antigen and the type III secretion system of *S. flexneri*, with some O-antigen-specific antibodies capable of cross-reacting against other serotypes. Interestingly, not all antibodies against the T3SS were created equal, with some antibodies inhibiting infection in vitro and others enhancing infection. These data offer a landscape of the *S. flexneri* antibody response and could guide future vaccines for this pathogen. –Courtney Malo

## One Sentence Summary

An outbreak of *Shigella* in nonhuman primates enabled discovery of over 100 protective and pathogenic antibodies against key vaccine antigens.
