# Supplementary material for: Multifunctional antibody responses from a primate Shigella outbreak inform vaccine design
Source: Sci Transl Med. Author manuscript; Available in PMC 2026 Oct 7. (PMC7619511; doi:10.1126/scitranslmed.aef7084)
Supplement: Supplementary file 1 [file EMS219129-supplement-Supplemental_Material.docx]

**Supplementary materials and methods**

**Nonhuman primate (NHP) maintenance and regulatory approvals**

NHPs were housed at the Wisconsin National Primate Research Center. Animal care and use were in accordance with the *Guide for the Care and Use of Laboratory Animals, (74)* the Animal Welfare Act, and protocols approved by the College of Letters and Science and Vice Chancellor for Research Institutional Animal Care and Use Committee of the University of Wisconsin-Madison. Animals were indoor housed on a 12-hour light/dark cycle with controlled temperature and humidity and were provided fresh water, commercially formulated monkey chow, fresh produce, and approved enrichment items daily. Animals were housed in groups, pairs, or singly when necessary, due to clinical, behavioral, or experimental reasons.

**Determination of NHP *Shigella* infection, bacterial isolation and characterization**

NHP *Shigella* infections were determined by culture. Testing was performed on feces, rectal/fecal swabs, colon tissue, or gingival swabs collected from either individual NHPs or as pooled enclosure samples. Diagnostic specimens were collected and submitted for enteric bacterial culture to the UW Veterinary Care, Microbiology Laboratory. Specimens were inoculated to Trypticase soy agar containing 5% sheep blood, Hektoen Enteric agar (HE), and Selenite enrichment broth (Remel) and incubated at 35 °C for 24 hours. The plates were examined for the presence of normal fecal microbiota and the HE agar was examined for typical non-lactose fermenting organisms resembling species of *Shigella*. The Selenite broth was subcultured to XLT4 media (Remel,) to screen for species of S*almonella.* Colonies resembling species of *Shigella* were subcultured to Trypticase soy broth containing 5% sheep blood and incubated for 24 hours at 35 °C. Identification and antimicrobial susceptibility testing were performed according to manufacturer recommendations (Vitek) for isolates resembling species of *Shigella.* Antimicrobial susceptibility methods and breakpoint interpretations were in accordance with Clinical Laboratory Standards Institute guidelines *(75)*. Confirmed *Shigella* isolates were then swabbed and placed into liquid Amies medium for transport. Following transport, isolates were plated on tryptic soy agar with Congo red, and single red colonies were saved in glycerol stocks at -80 °C. Classical serotyping was performed using *Shigella* antisera (Subgroup B, Key Scientific Products). Overnight cultures of each isolate were stained with crystal violet in 20% methanol, then washed with Dulbecco’s Phosphate Buffered Saline (DPBS). Suspensions of stained bacteria were gently mixed individually with the monovalent typing and grouping antisera and evaluated for agglutination and typed according to manufacturer’s key.

**Grading system for monitoring NHP symptoms**

Animal demographics and health information were collected from WNPRC’s Electronic Health Records system. Symptoms described in these records were tracked from symptom onset to resolution corresponding to the culture sample collection date and were scored from 0 to 5 based on the severity of symptoms. A score of 0 represented asymptomatic cases. A score of 1 to 4 was assigned based on the number of observations present from the following list: abnormal feces, lethargy/down in cage/quiet, dehydration, vomiting, weight loss/inappetence, and pallor. A score of 5 represented cases where either euthanasia was elected or the animal was found dead. Abnormal feces were defined as reports of one or more of the following: bloody feces, mucoid feces, watery feces, or diarrhea.

**Bacterial strains and culture conditions**

*Shigella* strains used for functional assays and Outer Membrane Vesicle (OMV) production in this study are listed in table S2. For functional assays, frozen bacteria from glycerol stocks were streaked on TSBA plates with 0.01% Congo Red overnight at 37 °C (with 5 μg/ml kanamycin for strains harboring a kanamycin resistance cassette). Single colonies were then picked and grown shaking in Tryptic Soy Broth (TSB) (BD Biosciences) broth at 30 °C overnight and subcultured 1:100 the following day and grown shaking at 37 °C.

**Bacterial genome sequencing and analysis**

Bacteria were grown overnight in TSB (BD Biosciences), and then genomic DNA was extracted using Wizard Genomic DNA Purification kit and quantified by Qubit dsDNA HS Assay kit. Genewiz (Azenta Life Sciences) conducted paired-end 150 bp Illumina sequencing on each sample. Galaxy USA suite of tools was used for genome analysis. Fastp *(78)* was used to filter raw sequencing reads, then BWA-MEM2 *(79)* was used to map these reads to a reference genome *(29)*. Bcftools consensus *(80)* was then used to create consensus sequences for each isolate. Variants were then called using snippy (<https://github.com/tseemann/snippy>) and these data were used to construct a phylogenetic tree with IQ-TREE *(81)*. Antimicrobial resistance genes were identified using AMRFinderPlus *(82)* in de novo assembled genomes constructed with Shovill (<https://github.com/tseemann/shovill>). The cladogram (Fig. 1B) was plotted using ggtree *(83)* and the phylogenetic tree (fig. S1A) was plotted using iTOL *(84)*.

**NHP serum, plasma, and PBMC isolation**

Blood was collected aseptically from NHPs into ethylenediaminetetraacetic acid (EDTA) and serum-separating (SST) vacutainer tubes. Blood in the SST tubes was allowed to clot, then centrifuged at 4000 x g for 10 min. The serum supernatant was collected and centrifuged at 14,800 x g for 10 min at 4 °C to remove cell debris, then collected and heat inactivated at 56 °C for 30 min. Uncoagulated blood from EDTA tubes was pooled by sample and diluted with an equal volume of 1X DPBS. The diluted blood was carefully floated on top of prepared Lymphoprep (STEMCELL) in 50 ml SepMate tubes (STEMCELL) and centrifuged at 1200 x g for 20 min with the brake off. The plasma and lymphocyte layers were transferred to separate tubes and spun down at 650 x g for 8 min to pellet lymphocytes. Plasma was harvested and heat inactivated at 56 °C for 30 min. The pellet was resuspended in ACK Lysis Buffer (Gibco) for 5 min, then quenched with an equal volume of 1X DPBS and centrifuged at 550 x g. After discarding the supernatant, the pellet was washed in 5 ml of 1X DPBS and spun down at 400 x g. Cells were resuspended to approximately 2 x 10^7^ cells/ml in cold fetal bovine serum (FBS). An equal volume of 20% dimethyl sulfoxide (DMSO) in FBS was added dropwise to cell suspension before freezing and storing in liquid nitrogen (LN_2_). Heat-inactivated plasma and serum were stored at -80 °C until thawing for use.

**Plasma cytokine determinations**

Plasma cytokines were quantified by manufacturer’s instructions with the LEGENDplex NHP Inflammation Panel. Data were collected using a BD FACSymphony and analyzed in FlowJo 10.10.0 (FlowJo LLC).

**Antigen preparation**

Production of IpaB has been described previously *(85, 86)*. Briefly, *ipaB* and *ipgC* (IpaB’s cognate chaperone) were cloned into pACYCDuet-1 with the IpaB being expressed with a His tag at the N-terminus and IpgC remaining untagged. The resulting plasmid was used to transform *E. coli* Tuner (DE3) for co-expression. The transformed bacteria were grown in Luria Broth (LB) containing chloramphenicol at 37 °C until A600 reached 0.8 at which protein expression was induced by addition of IPTG to 1 mM. After 3 h, bacteria were collected by centrifugation, washed and resuspended in IMAC binding buffer (20 mM Tris-HCl pH 7.9, 500 mM NaCl, 10 mM imidazole) with 0.1 mM AEBSF Protease Inhibitor and lysed using a microfluidizer at 18,000 psi with three passes. The cellular debris was removed by centrifugation at 10,000xg for 30 min and loaded onto a 5 ml HisTrap FF column. The IpaB/HT-IpgC was eluted with IMAC elution buffer (20 mM Tris-HCl pH 8.0, 500 mM NaCl, 500 mM imidazole), dialyzed into 50 mM Tris-HCl pH 8.0, and loaded onto a HiTrap Q FF column. The complex was eluted using a gradient of 50 mM Tris-HCl pH 8 containing 1M NaCl. Lauryldimethylamine oxide (LDAO) was then added to a final concentration of 0.1% to release the HT-IpgC. When the LDAO-treated L-DBF/HT-IpgC complex was passed over an IMAC column, the IpaB was collected in the flow-through with the HT-IpgC being retained on the IMAC column. Finally, IpaB was dialyzed into 20 mM phosphate, pH 7.2, with 150 mM NaCl (PBS) with 0.05% LDAO and stored at -80 °C. LPS levels were determined using a NexGen PTS with EndoSafe cartridges (Charles River Laboratories, Wilmington, MA). All proteins had LPS levels <5 Endotoxin units/mg.

IpaD, IpaB_28.226_, and mutagenized IpaD constructs (IpaD-A CS, IpaD-A SipD, IpaD-B CS, IpaD-B SipD, IpaD-NTerm CS, and IpaD-NTerm SipD) were cloned into a pET-28b(+) expression vector with N-terminal His-tags, and in some cases Avi-tags. These plasmids were transformed into competent BL21 *E. coli* (NEB) by heat shock and selected on agar plates with kanamycin. Single colonies were picked, incubated overnight in LB-media supplemented with kanamycin, and subcultured 1:100 into fresh Terrific Broth (TB) supplemented with kanamycin. After reaching an OD_600_ of 0.6, 1 mM isopropyl-β-D-thiogalactopyranosid (IPTG) (Sigma-Aldrich) was added, and bacteria were incubated for an additional 18 h with shaking at 18 °C. Subsequently, bacteria were centrifuged at 4000 x g for 20 min, resuspended in resuspension buffer (20 mM Tris-HCl pH 8, 150 mM NaCl, 2 mM MgCl_2_, 1:10,000 benzonase, and cOmplete EDTA-free protease inhibitor) then lysed with a probe sonicator. Recombinant protein was purified from the supernatant using HisPur Ni-NTA Resin (Thermo Fisher Scientific) by gravity-flow column. IpaB_28.226_ was purified by anion exchange chromatography followed by size exchange chromatography, and mutagenized IpaD constructs were purified by size exchange chromatography. All proteins were monomeric and monodisperse. Protein yields were measured using NanoDrop A280, and purity was assessed by SDS-PAGE and size exclusion chromatography (SEC).

OMVs were purified as previously described in Xerri *et al*. *(40)*. Briefly, supernatant was collected from *S. flexneri* 2a or 3a tolR::kan mutants grown overnight in *Shigella sonnei* defined media (SSDM) *(87)* supplemented with l-methionine and l-tryptophan, or an overnight culture of *S. flexneri* Y was sonicated 20 min in a bath sonicator at room temperature to induce OMV release. The supernatants were then filter-sterilized, concentrated by tangential flow filtration, and ultracentrifuged to pellet OMVs, which were then resuspended in PBS and stored at -20 °C. Total protein content of OMVs was quantified by Micro-BCA assay (Thermo Fisher Scientific) following the manufacturer’s instructions.

O-Antigen (O-Ag) was purified from whole bacteria as previously described *(40)*. In short, an overnight culture of *S. flexneri* Y was treated with acetic acid to hydrolyze the O-Ag. After hydrolysis, the culture was neutralized, bacterial debris was pelleted, the supernatant was buffer exchanged by tangential flow filtration (Sartorius) first into 1 M NaCl, and then into ddH2O. Citrate buffer (20 mM, pH 3) was added to the supernatant, incubated at room temperature (RT) for 30 min, debris was pelleted, and then the supernatant was passed through a Sartobind IEX S75 cationic exchange filter (Sartorius). After cation exchange, the supernatant was precipitated once more with 18 mM Na2HPO4, 24% ethanol, and 200 mM CaCl_2_ at room temperature for 30 min, then buffer exchanged as described above before being concentrated in ddH2O using a 30 kDa cutoff spin filter (Amicon). The purified O-Ag was quantified using a Total Carbohydrate Assay Kit (Sigma-Aldrich) following the manufacturer’s instructions. Contaminating DNA and protein levels were assessed by nanodrop analysis and Micro BCA assay, respectively, and confirmed to be at least 10-fold lower than carbohydrate concentration.

**Antigen ELISAs**

High-binding 96- or 384-well plates were coated with 5 µg/ml of protein antigen in DPBS (IpaD, IpaB, or ovalbumin), or with 2.5 µg/ml of OMVs in DPBS overnight at 4 °C. This solution was then discarded, and each well was blocked with 3% Bovine Serum Albumin (BSA), 0.1% Tween in DPBS for protein antigens and 3% BSA, 0.01% Tween in DPBS for OMVs at 37 °C for 1 h. Blocking solution was then discarded, and serial dilutions of serum or monkey IgG1 (Mk-IgG1) monoclonal antibody in DPBS were added before incubating at 37 °C for 1 h. Plates were then washed 3x with 0.05% Tween in DPBS for protein antigens and 0.001% Tween in DPBS for OMVs. After washing with DPBS-Tween, plates were incubated with AP-conjugated goat anti-monkey IgG antibody (Abcam, ab112765) for 45 min at room temperature. After plates were washed again 3x with the appropriate DPBS-Tween concentration, developing buffer (100 mM NaCl, 100 mM Tris-HCl pH 9.5, 50 mM MgCl_2_, 1% Tween 20, and 1 mg/ml phosphatase substrate) (Sigma-Aldrich) was added to each well. Plates were incubated for 20 min at room temperature before absorbance was measured at 405 nm using a BioTek Synergy multiplate reader.

**Serum Bactericidal Assay (SBA)**

SBA experiments were adapted from previously published protocols *(88)*. The desired *Shigella* strain was grown to mid-log phase in TSB (BD Biosciences), washed, and resuspended in SBA buffer (1X HBSS + 0.1% gelatin, pH 7.4). In a 96-well V-bottom plate, 3x10^4^ CFU of *S. flexneri* were combined with 7.5% baby rabbit complement (BRC, Pel Freez Biologicals), the desired serum or monoclonal antibody, and SBA buffer in a final volume of 100 µl. This 7.5% BRC concentration was determined in pilot experiments as the highest concentration of BRC that did not result in non-specific bacterial killing, thus conferring sufficient sensitivity to detect antibody complement coordination. Plates were incubated at 37 °C for 3 h, washed once with 100 µl of SBA buffer, then mixed 1:1 with BacTiter-Glo (Promega) in a white 96 well plate and incubated for 5 min at room temperature. Luminescence was detected on a BioTek Synergy plate reader. Each plate contained 6 wells of background buffer and BRC only controls, and 6 wells of bacteria with BRC but no antibody controls. Background was subtracted from every well, then each well was normalized as a percentage of the bacteria with BRC but no antibody control wells. The data were fit with four parameter logistic curves with top values constrained to 100 and bottom values constrained to 0 in GraphPad Prism.

**Cell culture**

THP-1 cells (ATCC, TIB-202) were cultured in RPMI complete media (RPMI-1640 Medium [Gibco], 4.5 g/L glucose, 1:100 GlutaMAX [Gibco], 10 mM HEPES [Gibco], 1 mM Sodium Pyruvate [Gibco], 55 µM beta-mercaptoethanol [Gibco], 10% fetal bovine serum [Omega Scientific], 50 U/ml penicillin and 50 µg/ml streptomycin [Gibco]). Cells were passaged by transfer to fresh media without washing every 2 to 3 days and maintained at a density of 1 x 10^5^ to 5 x 10^5^ cells/ml. HeLa cells (ATCC, CCL-2) were passaged every 3 to 4 days in DMEM complete media (DMEM [Gibco], 1:100 GlutaMAX [Gibco], 10% fetal bovine serum [Omega Scientific], 50 U/ml penicillin and 50 µg/ml streptomycin [Gibco]). 0.25% Trypsin-EDTA (Gibco) was used to dissociate adherent cells from flask surface. Expi293F cells were passaged by transfer to fresh media without washing every 3 to 4 days in Expi293 Expression Medium (Gibco) and grown to a density of 3 x 10^6^ to 5 x 10^6^ cells/ml. Expi cells were cultured in shaker flasks using a rotating incubator. MS40L-low cells *(89)* were passaged every 2 to 3 days in complete B cell media (RPMI-1640 Medium [Gibco], 1:100 GlutaMAX [Gibco], 10 mM HEPES [Gibco], 1 mM Sodium Pyruvate [Gibco], 1:100 MEM NEAA [Gibco], 55 µM beta-mercaptoethanol [Gibco], 10% fetal bovine serum [Omega Scientific], 50 U/ml penicillin and 50 µg/ml streptomycin [Gibco]).

**Opsonophagocytosis assay (OPA)**

The desired *Shigella* strain was grown to mid-log phase in TSB (BD Biosciences), washed with PBS, then stained with a 1:50 dilution of CellTrace Violet (CTV, Thermo Fisher Scientific) for 15 min at 37 °C. Excess dye was quenched with FACS buffer (PBS + 1% FBS). The bacteria were washed once in PBS and resuspended in SBA buffer (1X HBSS + 0.1% gelatin, pH 7.4). 2.5 x 10^6^ Colony Forming Units (CFUs) of bacteria were mixed with a 1:40 final dilution of either NHP serum or plasma, or 50 nM of antibody and incubated at 37 °C for 30 min. Opsonized bacteria were then mixed with 5 x 10^4^ undifferentiated THP-1 cells (MOI of 50) and incubated for 30 min at 37 °C in 5% CO_2_. After incubation, the OPA reactions were fixed with 4% PFA in PBS on ice for 30 min. Cells were then washed in PBS and resuspended in FACS buffer (PBS + 1% FBS) before quantification by flow cytometry using a FACSCelesta or FACSymphony (BD Biosciences) and analysis via FlowJo 10.10.0 (FlowJo LLC).

**Adhesion and Invasion Assay (AIA)**

One day before the assay, TC-treated 96-well plates were seeded with 4 x 10^4^ HeLa cells per well in 100 µl of DMEM complete media without antibiotics. The following day, *Shigella* was grown to mid-log phase in TSB (BD Biosciences), then stained with CTV as described in the OPA methodology. 20 µl of the bacteria resuspended to a concentration of 2.4 x 10^8^ CFU/ml was added to 96-well V-bottom plates pre-loaded with 5 µl of 1:10 dilutions of serum or plasma (final dilution of 1:50), then incubated at 37 °C for 30 min. 20 µl of this mixture was then transferred to each well of the prepared HeLa plate (MOI of 50) and centrifuged at 900xg for 10 min, then incubated at 37 °C for 30 min in 5% CO_2_. After incubation, cells were washed once with PBS, dissociated with Trypsin (Gibco), fixed with 100 µl of 4% paraformaldehyde (PFA) in PBS, then incubated on ice for 30 mins. Cells were then centrifuged at 400 x g for 5 min and resuspended in FACS buffer (PBS + 1% FBS). Samples were then quantified by flow cytometry using a FACSCelesta or FACSymphony (BD Biosciences) and analyzed using FlowJo 10.10.0 (FlowJo LLC). % Inhibition was calculated relative to the invasion rate of bacteria pre-incubated in PBS.

**Hemolysis assay**

The hemolysis assay was adapted from published studies *(90)*. The desired *S.* *flexneri* strains were grown to mid-log phase in TSB (BD Biosciences). For assays with *S. sonnei*, an O-antigen knockout strain was used to expose the T3SS needle tip, and these cultures were supplemented with kanamycin. Cultures were centrifuged, washed in PBS, and resuspended in PBS to a final concentration of 6.6 x 10^9^ CFU/ml. 45 µl of this suspension was added to each well of a 96-well V-bottom plate preloaded with 5 µl of 10 µM mAb or 5 µl of undiluted serum and incubated at 37 °C for 30 min. Approximately 1 ml of sheep red blood cells (RBCs, Innovative Research) were washed 3 times with PBS and resuspended to a concentration of 1 x 10^9^ RBC/ml. 50 µl of washed RBCs was added to each well of the plate, centrifuged for 5 mins at 2000 x g, and incubated at 37 °C for 30 min. The hemolysis reactions were then stopped by adding 100 µl of ice-cold PBS to each well and resuspending pellets with each addition. Plates were centrifuged again for 5 min at 2000 x g. 100 µl of supernatant was transferred from each well of the V-bottom plate to a new half-area 96 well plate. Plates were read at 545 nm on a BioTek Synergy multiplate reader. Each plate contained wells of RBCs with no bacteria to measure background and wells with bacteria and an isotype control antibody, Adalimumab *(91)*, to measure baseline hemolysis. Background values were subtracted from every well, then each well was normalized as a percentage of hemolysis from the wells containing Adalimumab. Rabbit polyclonal IpaB antibody was obtained from Cusabio (CSB-PA322404ZA01SZB).

**NHP B cell sorts**

Sorting of antigen-specific memory B cells was performed as previously described (citations, including that activation paper). Cryopreserved PBMCs were thawed and labeled with the following antibodies: CD3-APC-Cy7 (OKT3), CD4-APC-Cy7 (OKT4), CD8-APC-Cy7 (RPA-T8), CD14-APC-H7 (M5E2), CD16-APC-H7 (3G8), CD20-PerCP-Cy5.5 (2H7), IgM-BV785 (G20-127), and IgG-BV421 (G18-145), together with LIVE/DEAD Aqua Cell Stain in FACS buffer (PBS + 1% FBS). Cells were then stained with the antigen of interest (OMVs labeled with Vybrant DiO or DiD solutions [Thermo Fisher Scientific], IpaD labeled with biotin [Avidity BirA500] and coupled to streptavidin-AF488 and -AF647 [Thermo Fisher Scientific], or IpaB labeled with NHS esters of AF488 and AF647 [Thermo Fisher Scientific]). Because verified antigen-naïve controls were not available, antigen-positive gates were established using conservative fluorescence intensity thresholds informed by prior antigen-specific B cell sorting experience. Antigen specific memory B cells (CD3^-^, CD4^-^, CD8^-^, CD14^-^, CD16^-^, CD20^+^, IgM^-^, IgG^+^, antigen double positive) were sorted using a BD FACSMelody into individual wells of either an empty 96-well PCR plate or a 96-well cell culture plate pre-seeded with 3 x 10^3^ MS40-lo cells in 100 µl of complete B cell media supplemented with 5 µg/ml IL-2, 1 µg/ml IL-4, 1 µg/ml IL-21, and 1 µg/ml BAFF (PeproTech). PCR plates were immediately stored at -80 °C until PCR processing. Cell culture plates were maintained and passaged every 2 to 3 days to facilitate activation. 21 days post-sort, supernatants were assayed for antigen-specific binding by ELISA, and cells from positive wells were lysed in a buffer containing IGEPAL (Sigma-Aldrich), 0.1 M DTT and RNAseOUT (Thermo Fisher Scientific), transferred to a 96-well PCR plate, and stored at -80 °C until processing.

**Recombinant monoclonal antibody cloning and expression**

Monoclonal IgG heavy and light chain sequences were amplified from either sorted single cells or activated cells frozen in lysis buffer that were stored at -80 °C. SuperScript IV Reverse Transcriptase and Buffer (Thermo Fisher Scientific) was used for RT-PCR in a master mix containing RNaseOUT (Thermo Fisher Scientific), random hexamer mix, 0.1 M DTT, dNTPs, and reverse primers for heavy, kappa, and lambda chains. The resulting cDNA was amplified via an initial PCR reaction (PCR1) using HotStarTaq MasterMix (Qiagen) and primers for heavy and light chains. A second PCR reaction (PCR2) was performed on samples to add Gibson overhangs at the beginning and end of amplified antibody chain sequences, which used a master mix containing Phusion Enzyme (Thermo Fisher Scientific), HF Buffer (Thermo Fisher Scientific), dNTPs, MgCl_2_, and primers for heavy and light chains. PCR2 products were screened via addition of SYBR Safe DNA stain (Thermo Fisher Scientific) and imaging to see which PCR wells successfully amplified a heavy, kappa, or lambda chain sequence. PCR2 hits were re-arrayed and run on a 1% agarose gel. After excising bands from gel, antibody chain sequences were isolated via ZR-96 Zymoclean Gel DNA Recovery Kit (Zymo) and assembled into Mk-IgG1 expression vectors via HiFi DNA Assembly (NEB). Assembled plasmids were transformed into NEB 5-alpha competent cells (NEB) and plasmid DNA was purified via either Zyppy-96 Plasmid Kit (Zymo) or NucleoSpin Plasmid Transfection-grade miniprep kit (Macherey-Nagel). Plasmid DNA was purified with Zyppy kit for initial colony screening via Sanger sequencing, NucleoSpin kit was used to prepare final transfection-quality DNA. Expi293 Expression System was used for transfecting heavy and light chain antibody pairs. Transfections used a 1:2.5 heavy/light chain ratio by mass. FectoPRO transfection reagent (Avantor) was added to plasmid DNA diluted in Opti-MEM (Gibco) and incubated at room temperature for 10 min before Expi293F cells were added at a concentration of 3 x 10^6^/ml. Transfections were left to express in rotating incubator for 5 days. 24 h post-transfection, cells were fed a mix of 300 mM Valproic acid sodium salt (Sigma-Aldrich) and 45% glucose solution (Sigma-Aldrich). Expressed monoclonal antibodies were purified 5 days post-transfection by centrifuging cells and incubating sterile-filtered supernatant with Praesto AP+50 beads (Purolite) for 3 h at 4 °C. Resin was transferred to Polyprep columns (Bio-Rad) and washed 3x with PBS by gravity-flow. IgGs were eluted using Pierce IgG Elution Buffer (Thermo Fisher Scientific) and brought to a neutral pH with 1 M Tris-HCl pH 8.0 (Quality Biological). Antibody solutions were concentrated and buffer-exchanged into PBS using Amicon Ultra-15 30 kDa filter units (Sigma-Aldrich).

**O-antigen and IpaD-specific mAbs bacterial surface stains**

The desired *Shigella* strains were grown to mid-log phase in TSB (BD Biosciences). *S. flexneri* O-Ag- (to negatively screen for O-Ag-specific mAbs), *S. sonnei* lvp::kan (to stabilize the virulence plasmid for O-Ag-specific mAbs staining), and *S. sonnei* OAg::kan (to remove the O-Ag and expose the T3SS needle for Ipa mAb staining) were cultured in TSB supplemented with kanamycin.

For IpaD and OAg-specific antibodies, bacteria were pelleted, washed, and resuspended in PBS to a concentration of 2 x 10^9^ CFU/ml. In a 96-well V-bottom plate, 25 µl of bacteria were added to 25 µl of antibody at 2X final concentration and incubated at 37°C for 30 mins. The bacteria were then washed in PBS and resuspended in 25 µl of secondary antibody (BD Horizon™ BV421 Mouse Anti-Human IgG), diluted 1:100 in PBS and incubated for 30 min at room temperature. To fix the bacteria, 100 µl of 4% PFA in PBS was added to each well and the plates were incubated on ice for at least 30 min. Bacteria were pelleted and then resuspended in FACS buffer (PBS + 1% FBS). Fluorescence was read on a BD FACSCelesta or BD FACSymphony. Data were analyzed on FlowJo 10.10.0 (FlowJo LLC).

**O-Antigen visualization and western blotting**

To visualize O-Ag, crude protein-free lipopolysaccharide (LPS) extract was purified from *S. flexneri* Y, *S. flexneri* 3a, *S. flexneri* 3a waaL::kan (O-Ag-), or *S. flexneri* 3a waaL::kan pwaaL induced with 1 mM IPTG following the method of Darveau and Hancock *(92)*. Bacterial LPS extract was separated by SDS-PAGE. For total O-Ag visualization, the gels were stained with Pro-Q™ Emerald 300 Lipopolysaccharide Gel Stain Kit (Thermo Fisher Scientific) following the manufacturer’s instructions. For western blotting, gels were fixed with 5% acetic acid and 50% isopropanol, then washed with ultrapure water and stained with 50 nM antibody in antibody buffer (5% BSA, 0.1% SDS, 0.1% Tween 20 in PBS) overnight with gentle agitation. The gel was washed twice with PBS + 0.1% Tween 20, then stained for 2 hours with AF488 anti-human IgG (M1310G05, BioLegend) diluted 1:100, then washed twice. Stained gels were visualized on a ChemiDoc MP Imaging System (Bio-Rad).

**IpaB-specific mAbs bacterial surface stains**

*Shigella* strains were grown to mid-log phase in fresh TSB (BD Biosciences) supplemented with 0.1% deoxycholate (Sigma-Aldrich). In a 96-well V-bottom plate, 25 µl of unwashed bacterial culture was added to 25 µl of pre-warmed antibody at 2X final concentration, working quickly to avoid the culture cooling, and incubated at 37 °C for 30 min. 100 µl of ice cold 4% PFA in PBS was then directly added to the plate and incubated on ice for 15 min. Bacteria were washed with PBS and resuspended in 1:100 unconjugated mouse anti-human IgG antibody (G18-145, BD Biosciences) in PBS followed by a 30 min incubation at 37 °C. Bacteria were washed with PBS and resuspended in 1:100 AF488-conjugated goat anti-mouse IgG (Thermo Fisher Scientific) followed by another 30 min incubation at 37 °C. Bacteria were washed with PBS, resuspended in FACS buffer, and quantified on a BD FACSymphony. Data were analyzed on FlowJo 10.10.0 (FlowJo LLC).

**Antibody isotype switching / purifications**

Heavy and light chain sequences from representative mAbs were PCR-amplified from IgG1 plasmid DNA using primers with proper overhang sequences following the same Phusion protocol as our PCR2 cloning step. PCR products were run on a 1% agarose gel and gel extracted via ZR-96 Zymoclean Gel DNA Recovery Kit (Zymo). Heavy and light chain pairs were Gibson assembled into parent vectors to express antibodies in the form of IgG3 and monomeric IgA1 via HiFi DNA Assembly (NEB). Plasmid DNA was purified using Zyppy-96 Plasmid Kit (Zymo) and screened for correct assembly via Sanger sequencing. Sequenced plasmids were purified via NucleoSpin Plasmid Transfection-grade miniprep kit (Macherey-Nagel). Heavy and light chain pairs were transfected following the same transfection protocol as previously described, with the exception of monomeric IgA1 using a 1:1 heavy/light chain ratio. 5 days post-transfection, cells were centrifuged and supernatants were sterile-filtered and incubated for 3 h at 4 °C with either Protein A/G UltraLink Resin (Thermo Fisher Scientific) for IgG3s or Peptide M Agarose Resin (Invivogen) for IgA1s. Antibodies were purified and buffer-exchanged following the same column purification procedure as previously described. Purified IgG3 and IgA1 antibodies were run on a Mini-PROTEAN TGX 4–20% Precast Gel for size validation (Bio-Rad).

**Mice**

Mouse experiments were approved by the Office of Animal Research (OAR) and the institutional IACUC at the University of Missouri (Protocol: 38241). Six-to-eight-week-old female C57BL/6 mice (Charles River Laboratories, Wilmington, MA) were used in this study. Groups were as follows (n=10): isotypic control mAb VRC01 (negative control); mAb D13r.34, D02-E4, B07.24, B10.29. Mice received a single intranasal dose of antibody at 10 mg/kg in a total volume of 30 to 50 µl (based on the weight of each mouse). Antibody administration was performed 6 h prior to Shigella challenge. S. flexneri 2a 2457T challenge strains were grown on tryptic soy agar containing 0.025% Congo red at 37 °C overnight, subcultured in TSB on the challenge day to an A600 of 1.0, and diluted to 5 x 10^5^ CFU (LD_50_) in 30 µl PBS for intranasal (IN) challenge. Mice were monitored for two weeks, with euthanasia criteria including >25% weight loss or blood glucose ≤100 mg/dL. This experiment was conducted two independent times, and data were combined for analysis.

**PBMC stimulation assays**

Cryopreserved PBMCs were thawed and seeded into TC-treated 96 well plates at a density of 7 x 10^5^ cells per well in RPMI complete media (RPMI-1640 Medium [Gibco], 4.5 g/L glucose, 1:100 GlutaMAX [Gibco], 10 mM HEPES [Gibco], 1 mM Sodium Pyruvate [Gibco], 55 µM beta-mercaptoethanol [Gibco], 10% fetal bovine serum [Omega Scientific], 50 U/ml penicillin and 50 µg/ml streptomycin [Gibco]). The cells were rested for 3 h at 37 °C with 90% humidity and 5% CO_2_. CD40 blocking antibody (1 µg/ml final conc.) and stimulating antigen (Ipa proteins 10 µg/ml, SEB 5 µg/ml final conc.) were added to each well, bringing total volume per well to 200 µl. After 14 h of incubation, 100 µl of media was collected for cytokine determinations using the LEGENDplex NHP Th Cytokine Panel (BioLegend) according to the manufacturer’s instructions. 10 µl of Brefeldin pre-diluted 1:100 in RPMI complete media (final dilution 1:1,000) was then added. After 4 h more of incubation, cells were dissociated with Trypsin (Gibco) and transferred to 96-well V-bottom plates. Cells were stained in FACS buffer with the following antibodies: CD3-AF488 (SP34-2), CD4-BV421 (OKT4), CD8-APC-Cy7 (RPA-T8), NKG2A-Vio Bright V600 (REA110), OX40-PE-Cy7 (Ber-ACT35), and 4-1BB-APC (4B4-1), along with LIVE/DEAD Near-IR Cell Stain. Cells were then fixed in BD CytoFix, permeabilized in BD CytoPerm, and stained for intracellular cytokines with the antibodies IFN-γ-RB744 (B27), and IL-17A-BUV737 (N49-653). Cells were then resuspended in FACS buffer and quantified on a BD FACSymphony, then the data were analyzed using FlowJo 10.10.0 (FlowJo LLC).

**Competition ELISA**

384-well plates were coated with 5 µg/ml of each antibody in PBS to be assayed in duplicate, then incubated overnight at 4 °C. This liquid was then discarded, and plates were blocked with 3% BSA, 0.1% Tween in PBS at 37 °C for 1 h. While the plates were blocking, 150 µg/ml of competing mAb was pre-incubated with either 1 µg/ml IpaD in PBS or 60 µg/ml IpaB in TBS+LDAO 0.05% at 37 °C for 30 min. The blocking solution was then discarded, and the antigen-antibody complexes were then transferred to the blocked plates and incubated at 37 °C for 1 h. The plates were then washed 3 times with either PBS+0.05% Tween for IpaD ELISAs or TBS+0.05% LDAO for IpaB ELISAs. 1:1000 of AP-conjugated mouse anti-His antibody (SB194b, SouthernBiotech) diluted in either PBS for IpaD or TBS+0.05% LDAO for IpaB was added to each well and incubated at 37 °C for 30 min. The plates were washed another three times with the appropriate buffer, then AP developing buffer was added to each well and incubated for 25 min at room temperature. Absorbance at 405 nm was measured using a BioTek Synergy multiplate reader. Self-competing wells were subtracted from each antibody as background values, then each antibody combination was normalized as a percentage of the signal from the unblocked coating antibody. These data were then used to create a Pearson correlation matrix and plotted in heatmap form.

**Cryo-EM sample preparation and data collection**

All IpaD Fabs were prepared by papain digest. The IpaD–D02-F2–D02-E4 complex was generated by combining purified IpaD with a 1.5-fold molar excess of D02-F2 Fab, 1.5-fold molar excess of D02-E4 Fab, and 6-fold molar excess of a previously described anti-Fab nanobody (Nb) *(93)*. The complex was isolated using a Superdex 200 Increase (Cytiva) SEC column equilibrated in HEPES-buffered saline (HBS). The final complex was concentrated to an absorbance at 280 nm (A280) of 0.57. The IpaD–D13r.34 complex was generated similarly, except that a 1.5-fold molar excess of D13-34 Fab and 3-fold excess of the anti-Fab nanobody were incubated with IpaD prior to SEC, and the final sample was concentrated to an A280 of 0.4. UltrAuFoil grids with 1.2-μm diameter/1.3-μm spacing (quantifoil) were plasma cleaned for 30 sec. Grids were prepared using a Vitrobot Mark IV (Thermo Fisher Scientific) set to 6 °C and 100% humidity using 4 µl sample with a 10 s wait time and a 4 s blot time at force 15 (IpaD–D02-F2–D02-E4) or force 12 (IpaD–D13-34) before plunging into liquid ethane. Data for the IpaD–D02-F2–D02-E4 sample were collected on a Talos Arctica operating at 200 kV (Thermo Fisher Scientific) equipped with a K3 direct electron detector (Gatan) using Serial EM. For the IpaD–D13-34 sample, data were collected on a Titan Krios operated at 300 kV (Thermo Fisher Scientific) equipped with a Falcon4i direct electron detector and a Selectris Energy Filter using EPU. See table S1 for data collection parameters.

**Cryo-EM data processing and model building**

For the IpaD–D02-F2–D02-E4 dataset, motion correction and dose weighting, followed by patch contrast transfer function (CTF) estimation, blob picking, and patch motion correction were performed in CryoSPARC Live *(94, 95)*. A stack of 1,734,751 particles was picked using CryoSPARC Live Blob Picker. Particles were subjected to one round of 2D classification, *ab initio* reconstruction, and three rounds of 3D heterogeneous refinement that resulted in a stack of 82,992 particles. Non-uniform refinement *(96)* was used to generate a map that was subsequently used for template-based particle picking on de-noised micrographs. A total of 2,107,885 particles were picked and extracted with 2x Fourier cropping. After multiple rounds of 2D classification and 3D heterogeneous refinement, a particle stack of 114,304 was subjected to local motion correction and extracted without binning. Heterogenous refinement was repeated until the map quality no longer improved, leaving a particle stack of 74,015, which was reduced to 73,375 during the final round of non-uniform refinement with reference-based motion correction *(94)*.

For the IpaD–D13r.34 dataset, patch motion correction and dose weighting, followed by patch contrast transfer function (CTF) estimation was performed in CryoSPARC. Blob picker was used to generate an initial stack of 3,710,545 particles which were extracted with 2x Fourier cropping. Two rounds of 2D classification were used to reduce the particle stack to 1,367,409. A subset of 500,000 particles was used to generate four *Ab initio* models. After three rounds of 3D heterogeneous refinement, a stack of 167,838 particles was subjected to local motion correction and extraction without binning. Heterogenous refinement was repeated until the map quality no longer improved, leaving a particle stack of 26,038. Local CTF refinement and reference-based motion correction did not improve map quality. Masking the constant region of the antibody fragment and the anti-Fab nanobody did not improve the quality of the final map in either dataset, which was highest when using automatic mask generation in non-uniform refinement.

The coordinates were built manually in Coot and ISOLDE *(97, 98)*, using starting models generated from AlphaFold3 *(99)*. The anti-Fab nanobody and the constant region of the antibody fragments were not included in the models. The structures were refined using real-space refinement in Phenix *(100, 101)*. Structural biology software used in this project was compiled and configured by SBGrid *(102)*. Figures were generated using PyMOL and ChimeraX *(103)*.

**Biolayer interferometry (BLI) assays**

BLI assays were performed using polypropylene black 96-well microplates (Greiner) on an Octet R8 (Sartorius) instrument at 30 °C. All reagents were diluted with 10X kinetics buffer (0.1% BSA, 0.02% Tween 20 in 1XPBS). 8 µM of His-tagged protein was captured with HIS1K Biosensors (Sartorius) for 60 seconds and transferred to 10X kinetics buffer for 30 seconds to wash off unbound protein. Sensors were then transferred into 30 nM of monoclonal antibodies for 60 seconds. The percent wildtype (WT) shift for each mAb was calculated with the formula: Percent WT shift (%) = (IpaD construct shift @ 60s [nm]) / (WT IpaD shift @ 60s [nm]) * 100.

**Fig. S1. Bacterial phylogeny and antimicrobial resistance.**

(**A**) Unrooted phylogenetic tree of the same strains shown in Fig. 1B. Branch lengths are proportional to evolutionary distance. (**B**) Heatmap of serotype, phylogenetic branch, antibiotic resistance profiles, and detected antibiotic resistance genes for each strain. AMP = ampicillin (MIC ≥ 32 µg/ml = resistant); AMX = amoxicillin (MIC = 16 µg/ml = intermediate); CEX = cephalexin (MIC ≥ 8 µg/ml = resistant); CAM = chloramphenicol (MIC ≥ 64 µg/ml = resistant); DOX = doxycycline (MIC = 16 µg/ml = resistant). (**C**) Alignment of gtrII translations alongside heatmap of serotype. Residue disagreements with reference sequence are highlighted in color, stop codons are black.

**Fig. S2. NHP clinical outcomes and serology.**

(**A**) Symptom index by infecting serotype (n = 1 to 109). Data were analyzed by one-way ANOVA with Tukey’s multiple comparisons test. Data are presented as mean ± SEM. (**B**) Symptom index by sex (n=68 males, n=91 females). Data were analyzed by an unpaired t-test with Welch’s correction. Data are presented as mean ± SEM. (**C**) Correlation of symptom index and age (n=158). Data were analyzed by simple linear regression, with an extra sum-of-squares F Test to determine whether the slopes significantly differ from 0. Data are presented with 95% confidence interval. (**D**) Cytokine levels of naïve (n=8) and 1-week post detection of infection plasma (n=41). Data presented as geometric mean ± geometric SD, analyzed by multiple lognormal Welch’s t-tests with Holm-Šídák correction. Experiment was conducted in technical duplicate. (**E**) IgG and IgM ELISA EC_50_ values against ovalbumin; samples > 40 were excluded from ELISA titer analyses as polyreactive. (**F** and **G**) Time-course of serum IgG (**F**) and IgM (**G**) ELISA EC_50_ values from Y-infected NHPs against IpaB, IpaD, and Y OMVs (n=46). Data are presented as mean ± SEM. (**H**) Ratio of IgM / IgG EC_50_ values from (E-F) (n=46). Data are presented as mean ± SEM. (**I**) SBA curves corresponding to the serum bactericidal assay IC_50_ values plotted in Fig. 1E. Data were analyzed by four parameter logistic curves, and are presented as mean ± SEM. Experiment was conducted in technical duplicate. (**J**) Representative flow plots of opsonophagocytosis assays using naïve and convalescent plasma. THP-1 cells are identified by size, then cells that have internalized bacteria are identified by CellTrace Violet signal (CTV^+^). (**K**) Representative flow plots of adhesion and invasion assays using naïve and convalescent plasma. HeLa cells are identified by size, then the extent to which bacteria have adhered/invaded is measured by % of cells that are CTV^+^. **p*< 0.05, ****p*< 0.001, and *****p*< 0.0001.

**Fig. S3. O-Ag mAb characterization.**

(**A**) SDS-PAGE of crude LPS extracts from *S. flexneri* Y, *S. flexneri* 3a, *S. flexneri* 3a waaL::kan (O-Ag-), and complemented *S. flexneri* 3a waaL::kan pwaaL stained with Pro-Q Emerald 3000. (**B**) Western blot of crude LPS extracts from the same samples as panel 3C probed with Y01.40. (**C**) Same data as Fig. 2G. Mutation frequencies and CDR3 lengths of OAg-specific mAbs by NHP. (n=5-34 mAbs per NHP) Data are presented as mean ± SEM, and analyzed by ordinary one-way ANOVAs with Tukey’s post-test. (**D**) Representative flow plots of bacterial surface stains against *S. flexneri* Y, 1b, 2a, 2b, 3a, 3b, 5a, 6, OAg^-^, and *S. sonnei*. **p*< 0.05, ***p<0.01*, ****p*< 0.001, and *****p*< 0.0001.

**Fig. S4. Functional correlations of O-Ag mAbs.**

(**A** to **D**) Correlations per serotype between surface stain and SBA IC_50_ values (**A**), surface stain and OPA activity (**B**), surface stain and hemolysis activity (**C**), and OPA activity and hemolysis activity (**D**). Each data point represents a mAb. mAbs that had no detectable SBA activity were assigned an SBA IC_50_ of 1000nM. Log- or semi-log best fit lines, Extra sum-of-squares F Test to determine whether the slope significantly differs from 0. For (**A**), data were analyzed with a log-log best fit line. For (**B and C**), data were analyzed with semi-log best fit lines. For (**D**), data were analyzed by simple linear regression. All data were analyzed with extra sum-of-squares F Tests to determine whether the slopes significantly differ from 0.

**Fig. S5. PBMC stimulation with IpaD and IpaB.**

(**A**) Schematic of experimental approach (graphics provided by NIH BioART). Cryopreserved NHP PBMCs collected two-weeks post detection of infection were thawed, rested 3 h, and stimulated with IpaD (10 µg/ml), IpaB (10 µg/ml), or SEB (5 µg/ml) in the presence of anti-CD40 antibody. After 14 h, cytokines in media were quantified by multiplex assay. Brefeldin A was then added to block cytokine secretion. After 4 hours, the PBMCs were harvested and stained for flow cytometry analysis. The experiment was conducted in technical duplicate. (**B**) Concentrations of IFN-γ, IL-17A, IL-21, IL-10, IL-6, TNFα, IL-2, IL-13, IL-4, and IL-5 detected in media collected following PBMC stimulations. Data are presented as geometric mean ± geometric SD, data were analyzed by one-way ANOVAs with Dunnett’s multiple comparisons tests. (**C**) Gating strategy for NK cells, NKT cells, CD4 T cells, CD8 T cells, and AIM^+^ (T cells OX40^+^ 4-1BB^+^). (**D**) Percent of total CD4 and CD8 T cells positive for AIM markers. Data are presented as geometric mean ± geometric SD, data were analyzed by one-way ANOVAs with Dunnett’s multiple comparisons tests. (**E**) Gating strategy for IFN-γ^+^ and IL-17A^+^ CD4 T cells. (**F**) Percent of total CD4, CD8, NK, and NKT cells positive for IFN-γ and IL-17A. Samples with 0% positivity were assigned the lowest detected value. Data are presented as geometric mean, data were analyzed by one-way ANOVAs with Dunnett’s multiple comparisons tests. **p*< 0.05, ***p<0.01*, ****p*< 0.001, and *****p*< 0.0001

**Fig. S6. Characterization of T3SS mAbs.**

(**A** and **B**) ELISA curves of Ipa-specific mAbs against IpaD or IpaB (**A**) and Ovalbumin (**B**). Data are presented as mean ± SEM. Data were analyzed by four parameter logistic curves. (**C**) Same data as Fig. 4G. Mutation frequencies and CDR3 lengths of Ipa-specific mAbs by NHP. (n=1-8 mAbs per NHP) Data are presented as mean ± SEM. Data were analyzed by ordinary one-way ANOVAs with Tukey’s post-test. (**D**) Representative flow plots of bacterial surface stains against *S. flexneri* 5a. (**E**) Percent hemolysis relative to isotype control of polyclonal IpaB antibodies against *S. flexneri* 2a (n=4-6 per condition, two independent experiments combined). Data are presented as mean ± SEM. Data were analyzed by unpaired t-test with Welch’s correction. (**F**) SBA curves for each Ipa-specific Mk-IgG1 mAb against *S. flexneri* Y. (**G** and **H**) Opsonophagocytosis assay with Ipa-specific Mk-IgG1 mAbs (**G**) or isotype-switched Hu-IgA1 and Hu-IgG3 mAbs (**H**). Each value is the percent of all CTV^+^ THP-1 cells. (**I**) Log rank (Mantel-Cox) test p-values for all pairwise comparisons of survival data plotted in Fig. 4I.

**Fig. S7. T3SS mAbs epitope mapping.**

(**A**) ELISA curves of IpaB-specific mAbs against IpaB_28.226_. Data are plotted as mean ±SEM. Data were analyzed by four parameter logistic curve fits. (**B**) Model of an IpaD monomer (PDB 2J0O) with the N-terminal domain, coiled-coil domain, and distal domains colored blue, gray, and red, respectively.

**Fig. S8. Cryo-EM data processing for the IpaD–D02-F2 Fab–D02-E4 Fab–anti-Fab Nb dataset.**

(**A**) Size-exclusion chromatography (SEC) trace for the IpaD–D02-F2 Fab–D02-E4 Fab–anti-Fab Nb complex shows clear separation of the complex from excess Fab-Nb complexes and excess free Nb. (**B**) Processing scheme used in CryoSPARC to determine the structure, including the final sharpened map and gold-standard Fourier shell correlation (FSC) curve calculated in cryoSPARC after FSC mask auto-tightening. The resolution was determined at FSC = 0.143 (horizontal black line). The final corrected mask gave an overall resolution of 3.4 Å.

**Fig. S9. Cryo-EM data processing for the IpaD–D13-34 Fab–anti-Fab Nb dataset.**

(**A**) Size-exclusion chromatography (SEC) trace for the IpaD–D13-34 Fab–anti-Fab Nb complex shows clear separation of the complex from excess Fab-Nb complex and excess free Nb. (**B**) Processing scheme used in CryoSPARC to determine the complex structure, including the final sharpened map and gold-standard Fourier shell correlation (FSC) curve calculated in cryoSPARC after FSC mask auto-tightening. The resolution was determined at FSC = 0.143 (horizontal black line). The final corrected mask gave an overall resolution of 3.3 Å.

**Fig. S10. IpaD antibody epitopes.**

(**A**) Schematic depicting the primary and secondary structure of IpaD, with the approximate sites of distal domain epitope A, distal domain epitope B, and the N-terminal domain epitope, as well as the N-terminal, coiled-coil, and distal domains, indicated (**B**) Model of an IpaD monomer (PBD 2J0O) with antibody epitopes identified in this study. D13r.34 is blue, D02-F2 is green, and D02-E4 is orange. (**C**) Model of IpaD monomers (PBD 2J0O) with previously discovered nanobody epitopes as determined by x-ray crystallography from Barta *et al*., 2017 (PDB 5VXJ, 5VXL, & 5VXM) (*62*). 20ipaD is red, JMK-E3 is yellow, and JPS-G3 is magenta. (**D**) Model of an IpaD monomer (PDB 2J0O) with previously discovered antibody epitope as determined by deep mutational scanning (DSM) associated with yeast surface display (YSD) (*58*).

**Fig. S11. Epitope-specific IpaD mutagenesis.**

(**A**) Amino acid sequence of each mutagenized IpaD construct aligned to wild-type IpaD. (**B**) Biolayer interferometry traces of IpaD-specific antibodies binding to wildtype IpaD loaded onto anti-HIS1K sensors. (**C**) Biolayer interferometry traces of IpaD-specific antibodies binding to distal domain epitope A mutant IpaD constructs loaded onto anti-HIS1K sensors. (**D**) Biolayer interferometry traces of IpaD-specific antibodies binding to distal domain epitope B mutant IpaD constructs loaded onto anti-HIS1K sensors. (**E**) Biolayer interferometry traces of IpaD-specific antibodies binding to N-terminal domain epitope mutant IpaD constructs loaded onto anti-HIS1K sensors. Gray traces indicate the antigen demonstrated >75% of WT binding, orange indicates 0-25% WT binding, and red indicates <0% WT binding, the same bins as in Fig. 6F.

**Table S1. Cryo-EM data collection, refinement, and validation statistics.**

|  | *IpaD–D02-F2 Fab–D02-E4 Fab–anti-Fab Nb* | *IpaD–D13r.34 Fab–*  *anti-Fab Nb* |
| --- | --- | --- |
| **Data collection and processing** |  |  |
| Microscope | Talos Arctica | Titan Krios |
| Magnification | 36,000 | 165,000 |
| Voltage (kV) | 200 | 300 |
| Electron exposure (e-/Å^2^) | 51.3 | 51.6 |
| Defocus range (μm) | ~1.5-3.0 | ~1.0-2.0 |
| Pixel size (Å) | 1.1 | 0.736 |
| Symmetry imposed | C1 | C1 |
| Initial particle images (no.) | 2,107,885 | 3,710,545 |
| Final particle images (no.) | 73,375 | 26,038 |
| Map resolution (Å)  FSC threshold | 3.4  0.143 | 3.3  0.143 |
|  |  |  |
| **Refinement** |  |  |
| Model composition  Non-H atoms  Protein residues  Ligands | 5,036  656  0 | 3,435  443  0 |
| Model vs. data CC  Overall  For ligands | 0.72  n/a | 0.84  n/a |
| R.m.s. deviations  Bond lengths (Å)  Bond angles (°) | 0.006  1.359 | 0.005  1.057 |
| Validation  MolProbity score  Clashscore  Poor rotamers (%) | 1.43  3.91  0 | 1.16  3.66  0 |
| Ramachandran plot  Favored (%)  Allowed (%)  Disallowed (%) | 96.25  3.75  0 | 99.07  0.93  0 |

**Table S2. Bacteria strains used in this study.**

| Strain name | Species | Serotype | Origin |
| --- | --- | --- | --- |
| S022 | *flexneri* | Y | This study |
| 210059 | *flexneri* | 1b | Gift from F. Fang *(28)* |
| 2457T | *flexneri* | 2a | ATCC 700930 |
| CDC 3591-52 | *flexneri* | 2b | ATCC 12022 |
| J17B | *flexneri* | 3a | ATCC 24570 |
| S012 | *flexneri* | 3b | This study |
| M90T | *flexneri* | 5a | Gift from E. Barry *(76)* |
| CCH060 | *flexneri* | 6 | Gift from E. Barry |
| S. flexneri ΔwaaL (O-Ag knockout) | *flexneri* | N/A | Xerri *et al*. *(40)* |
| S. flexneri ΔwaaL pwaaL (O-Ag complemented) | *flexneri* | 3a | Xerri *et al*. *(40)* |
| J17B tolR::kanR | *flexneri* | 3a | Xerri *et al*. *(40)* |
| 2457T tolR::kanR | *flexneri* | 2a | Xerri *et al*. *(40)* |
| 53G Lvp::KanR | *sonnei* | N/A | Gift from A. Clements *(77)* |
| 53G OAg::KanR | *sonnei* | N/A | Gift from A. Clements *(77)* |

Data file S1. Individual-level data
